# Fertilization and Cleavage Axes Differ In Primates Conceived By Conventional (IVF) Versus Intracytoplasmic Sperm Injection (ICSI)

**DOI:** 10.1038/s41598-019-51815-4

**Published:** 2019-10-25

**Authors:** Calvin R. Simerly, Diana Takahashi, Ethan Jacoby, Carlos Castro, Carrie Hartnett, Laura Hewitson, Christopher Navara, Gerald Schatten

**Affiliations:** 1 Pittsburgh Development Center, Division of Developmental & Regenerative Medicine, and Obstetrics-Gynecology-Reproductive Sciences, Cell Biology, and Bioengineering, University of Pittsburgh Cancer Institute, University of Pittsburgh School of Medicine, 204 Craft Avenue Pittsburgh, Pennsylvania, 15213 USA; 2 Division of Cardiometabolic Health, Oregon National Primate Research Center, Oregon Health & Science University, Beaverton, Oregon, 97006 USA; 3 CCRM Houston Main Center Memorial City, 929 Gessner Rd, Suite 2300, Houston, Texas 77024 USA; 4 The Johnson Center for Child Health and Development, Austin, Texas 78701 USA; 5 Department of Biology, South Texas Center for Emerging Infectious Disease, University of Texas at San Antonio, San Antonio, Texas 78249 USA

**Keywords:** Embryology, Mitosis, Centrosome

## Abstract

With nearly ten million babies conceived globally, using assisted reproductive technologies, fundamental questions remain; e.g., How do the sperm and egg DNA unite? Does ICSI have consequences that IVF does not? Here, pronuclear and mitotic events in nonhuman primate zygotes leading to the establishment of polarity are investigated by multidimensional time-lapse video microscopy and immunocytochemistry. Multiplane videos after ICSI show atypical sperm head displacement beneath the oocyte cortex and eccentric para-tangential pronuclear alignment compared to IVF zygotes. Neither fertilization procedure generates incorporation cones. At first interphase, apposed pronuclei align obliquely to the animal-vegetal axis after ICSI, with asymmetric furrows assembling from the male pronucleus. Furrows form within 30° of the animal pole, but typically, not through the ICSI injection site. Membrane flow drives polar bodies and the ICSI site into the furrow. Mitotic spindle imaging suggests para-tangential pronuclear orientation, which initiates random spindle axes and minimal spindle:cortex interactions. Parthenogenetic pronuclei drift centripetally and assemble astral spindles lacking cortical interactions, leading to random furrows through the animal pole. Conversely, androgenotes display cortex-only pronuclear interactions mimicking ICSI. First cleavage axis determination in primates involves dynamic cortex-microtubule interactions among male pronuclei, centrosomal microtubules, and the animal pole, but not the ICSI site.

## Introduction

With perhaps ten million ART babies now, fundamental problems regarding the mechanisms of fertilization and the onset of early development remain. Foremost among these are questions regarding which events are essential for the union of sperm and egg DNA within the activated zygote, as well as the consequences of different ART approaches, especially IVF as compared to ICSI, on the resultant embryo^1–6^. Notwithstanding the impressive literature on murine reproduction^7–11^, many differences between rodents and primates, especially in centriole and centrosome transmission^12–15^, preclude relevant conclusions. While human zygotes are investigated noninvasively, especially with the adoption of commercial time-lapse imaging systems, the earliest events during fertilization may not be captured in these systems, particularly during conception by ICSI. While research on animal models is increasingly regulated and has even been abandoned by some institutions (e.g., the UK’s historic Sanger Institute^16^), responsible research, especially research mimicking the noninvasive procedures employed on properly informed consenting couples undergoing infertility therapy, holds real promise for understanding basic principles during conception.

The establishment of embryonic axes that assemble the body’s three-dimensional plan, crucial for body patterning, implantation, and perhaps, embryonic stem cell derivations (including therapeutic cloning), are not well understood in most mammals^17–19^. Features defining undeniable oocyte polarity in mammals, as observed in some vertebrate (e.g., frogs) and invertebrate eggs, were largely lacking until pioneering work from several laboratories provided compelling evidence that ingrained polarities in mouse oocytes, before and after fertilization, track closely with features of the mouse blastocyst, the first stage with clearly morphologically identifiable polarity^20–24^. Specifically, the first cleavage plane predictably aligned orthogonally to the embryonic-abembryonic axes of the bilaterally symmetrical blastocyst^25^. The second polar body (2^nd^ PB; the product of the maturation of an egg cell during chromosomal reductional divisions) is the fiduciary mark of the animal pole in the mouse zygote and marking experiments of this site have demonstrated a strong correlation between the first division plane and the animal-vegetal (A → V) pole^20,26,27^. The sperm-induced fertilization cone may also be an accurate determinant of the cleavage plane^28–31^. Other experiments suggest cell shape, especially as influenced by the zona pellucida, is perhaps the most determinative factor for cleavage plane orientation^32^. Time-lapse video microscopy (TLVM) studies demonstrate how a pronounced oocyte shape change accompanying sperm incorporation mediated by actin influences the first cleavage axis, with the furrow passing through the short axis of the zygote and the site of sperm incorporation^33^. However, others question the assertion that the early mouse egg displays any predetermined axes. The orientation of the apposed male and female pronuclei was the most accurate predictor of the first cleavage plane since this axis defines the first mitotic spindle apparatus alignment, with cytokinesis occurring orthogonally to the aligned metaphase chromosomes^34,35^.

In humans, understanding zygotic polarity and embryonic axes relates to how best to select premier embryos at Day 3 or 5 for implantation and infertility management^36^. High-resolution time-lapse cinematography or the EmbryoScope™ TLVM system have been utilized to investigate both ICSI- and IVF-derived zygotes^37–41^. The highest quality embryos are derived from human IVF or ICSI zygotes with polarized nucleoli that tend to divide early, while poorer quality human zygotes appear to have a significantly higher variation between pronuclear alignment and the 2^nd^ PB^42–49^. In a retrospective time-lapse analysis of human ICSI zygotes, 95% underwent a meridional first cleavage division, demonstrating tetrahedral orientation at the 4-cell stage, predictive of the highest quality blastocysts for cyropreservation/embryo transfer^50^. Other TLVM human studies describe transient fertilization cone assembly (albeit at low frequency), pronuclei migration rates of 50 µm/hr, and varying juxtapositioned pronuclei within the zygote’s cytoplasm, but how these observations influence overall embryo quality is not yet known^41^. Ironically, in the rhesus monkey, an animal model closely aligned to human fertilization events^13^, fluorescent bead marking of ICSI sperm injection sites suggests that the sperm entry point (SEP) oriented the first cleavage plane and later aligned along the embryonic-abembryonic boundary in the blastocyst, as described for mice^30^.

To that end, this report presents findings on fertilization and early development in two primate models, macaques and baboons, with implications for understanding the still mysterious processes of human fertilization and the establishment of embryonic polarity.

Notwithstanding the impressive discoveries made by studying mice, clinically relevant findings rely upon studies on primates – human and nonhuman, alike^13^. Here, we have asked how similar ICSI is to conventional IVF. ICSI is perhaps the most used procedure in ART clinics; it is utilized extensively even in the absence of a defined male factor issue^51^. Consequently, this investigation focuses on fertilization and early developmental events in nonhuman primate (NHP) models to more accurately examine the events of human fertilization and how they might influence axis specifications with more clinical relevance.

## Results

### IVF- and ICSI-Fertilized zygotes differ in the cytoplasmic pronuclear orientation patterns after migration

**Figure 1:**
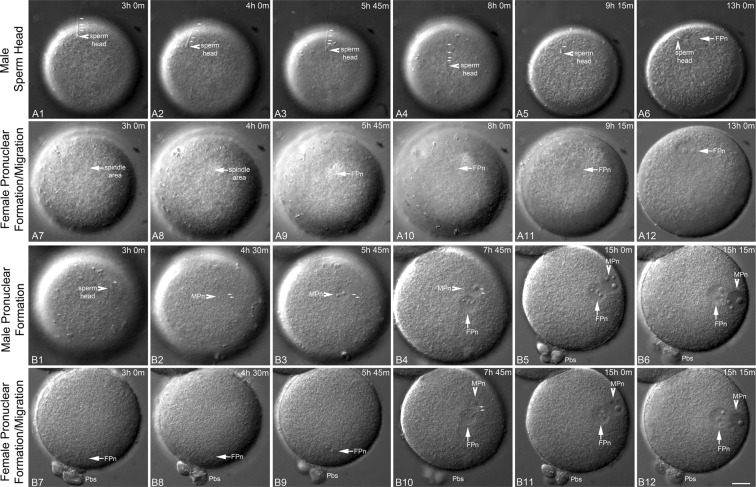
Selected TLVM panels of Rhesus sperm incorporation and pronuclear apposition during IVF. (**A1**–**A6**): male sperm head events during IVF (arrowheads) showing incorporation (**A1**,**A2**) followed by cortical rotation, first toward the site of the oocyte’s spindle region (**A3**–**A4**) and subsequently to a distal cortical site (**A5**–**A6**). The sperm head shows firm attachment to the oocyte’s cortex during penetration, even as cortical rotation powers sperm translocation. The sperm head is delayed in conversion to a fully decondensed MPn. (**A7**–**A12**): corresponding image panels from the TLVM video showing the position of the second meiotic spindle (**A7**–**A8**, arrows), FPn formation (**A9**–**A11**, arrows) and migration to the cortically residing sperm head (A12, arrow), mediated by the assembled sperm aster. Extrusion of the 2^nd^ PB is not visible in these panels. (**B1**–**B12**). Classical rhesus IVF and pronuclear apposition beginning 3 hrs post-insemination show sperm head plasma membrane penetration (**B1**, arrowhead), MPn formation (**B2**–**B3**, arrowheads), male and female pronuclear apposition (**B4**: MPn, arrowhead; FPn, arrow), and typical radial cortical parental genome orientation by the middle of interphase, with the MPn closest to the oocyte’s cortex (**B5**–**B6**, arrowheads) and the female distal within the cytoplasm (B5-B6, arrows). Note the orientation of the apposed pronuclei is oblique to the A → V axis. (**B7**–**B12**): corresponding images of FPn events. After 2^nd^ PB extrusion by 3 hrs post-insemination, the FPn forms near the cortex (**B7**–**B9**, arrows), swiftly migrates to the MPn utilizing microtubules of the assembled sperm aster (**B10**–**B11**, arrows), and ultimately positions distal to the cortically residing MPn (**B12**; FPn: arrow; MPn: arrowhead). The small arrows trace the sperm axoneme in (**A1**–**A5**,**B1**–**B4)**. Time is given in hours:minutes (h:m) post-IVF. All panels represent selected HMC images from a TLVM recording. Bar = 20 µm.

**Figure 2:**
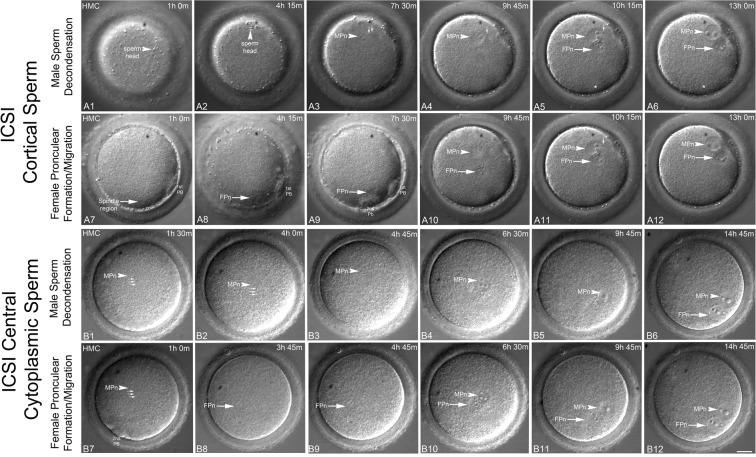
Selected TLVM panels of NHP sperm head decondensation, pronuclear assembly, migration and orientation following ICSI. (**A1**–**A12**). FPn and MPn formation, apposition, and cytoplasmic orientation by mid-interphase. Following ICSI, the cortically residing sperm head is visible translocating along the cortex, probably by cytoplasmic flow (**A1**–**A2**, arrowheads). Sperm head decondensation into an MPn is observed cortically (**A3**–**A4**, arrowheads) as the sperm aster assembles and promotes FPn migration (**A5**: MPn, arrowhead; FPn, arrow). Apposed FPn and MPn are oriented para-tangentially to the overlying cortex and obliquely to the A → V axis (**A6**: MPn, arrowhead; FPn, arrow; 2^nd^ PB position out of focal plane marked with *). FPn formation following elicitation of the second polar (2^nd^ PB) is observed in (**A7**–**A9)** (arrows) with migration to the cortical MPn and tangential positioning at the cortex seen in **A10–A12** (arrows; FPn, female pronucleus; MPn, male pronucleus). (**B1**–**B12**). FPn and MPn events after sperm deposition in the central cytoplasm. After central cytoplasmic sperm deposition by ICSI, the sperm head undergoes decondensation (**B1-B2**, arrowheads; small arrows, incorporated sperm axoneme) as the developing MPn jostles within the central cytoplasm (**B2-B4**; arrowheads). The FPn migrates along sperm astral microtubules to arrive at the MPn (**B4**, arrowhead) and the apposed parental genomes translocate to the oolemma cortex, aligning para-tangentially to the zygote surface and obliquely to the A → V axis (2^nd^ PB in **B7**). FPn forms after 2^nd^ PB formation (**B7**–**B9**: arrows) and completes migration to the decondensed MPn by 6.5 hr post insemination (**B10**: FPN, arrow; MPn, arrowhead). Over the next 8 hrs, the apposed parental genomes translocate to the cortex (**B11-B12**; FPn, arrow; MPn, arrowhead). The small arrows trace the sperm axoneme in (**A3**–**A5**,**A11**,**B1**,**B2**,**B7)**. Time is given in hours:minutes (h:m) post ICSI. All panels represent selected HMC images from a TLVM recording. Bar = 20 µm.

To investigate early pronuclear events in IVF- and ICSI-fertilized zygotes, we performed multidimensional TLVM recordings after fertilization (Figs 1 and 2; Supplemental Videos 1–3). A fused, anchored sperm head, bound tightly to the oolemma by 3 hrs post-IVF, is depicted in Fig. 1A1–A6. Cortical rotation, first toward the site of the arrested second meiotic spindle, followed by a gradual return to a distal cortical site, was indictive of the oocyte shape changes during the first 13 hrs post-insemination (Supplemental Videos 1). A female pronucleus (FPn) formed after oocyte activation, eventually moving toward the cortically residing sperm head (Fig. 1A7–A12), presumably along a microtubule-based sperm aster assembled from the reconstituted centrosomes^12,52,53^. No sperm incorporation cone assembled, though cortical streaming was evident (Supplemental Video 1). A second IVF-inseminated rhesus TLVM recording showed that the male pronucleus (MPn) formed at the oolemma sperm penetration site (Fig. 1B1–B6), with little cortical rotation, but with cytoplasmic contraction-expansion cycles observed (Supplemental Video 2). FPn migration to the cortical MPn measured 0.387 ± 0.056 µm min^−1^ (n = 5; Fig. 1B7–B12), with all zygotes showing radially apposed pronuclei relative to the adjacent overlying cortex and 80% aligning obliquely to the A → V axis by late interphase (Table 1; Supplemental Fig. S1).

**Table 1:** NHP Pronuclear Migration, Cortical Alignment and Orientation to Zygotic Axes Analyzed from TLVM Recordings.

Insemination type	Total n scored	Oocyte insemination position	Mean ± S.E. rate of ♀ PN migration to ♂ PN in µm min^−1^ [n]	Pronuclear Orientation to Oolemma (%)		Pronuclear Alignment Relative to Oocyte Axis at NEBD (%)		
				radial	Para-tangential	Meridional axis (A → V)	Equatorial axis	Oblique axis
IVF	5	cortical	0.387 ± 0.056 [5]	5/5 (100)	0	0	1/5 (20)	4/5 (80)
ICSI	52	cortical	0.539 ± 0.044^a,b^ [35]	22/52 (42)	30/52 (58)	3/52 (6)	4/52 (8)	45/52 (86)
ICSI	4	central	0.688 ± 0.118^c^ [4]	1/4 (25)	3/4 (75)	0	1/4 (25)	3/4 (75)

^a^female pronuclear migrate rate not significantly different between ICSI cortical versus IVF cortical sperm (p < 0.27).

^b^female pronuclear migration rate not significantly different between ICSI cortical versus ICSI central sperm (p < 0.61).

^c^female pronuclear migration rate not significantly different between ICSI central versus IVF cortical sperm (p < 0.16).

Rhesus ICSI oocyte TLVM recordings are presented in Fig. 2 and Supplemental Video 3. An immobilized spermatozoon microinjected near the cortex showed significant cytoplasmic sperm head displacement compared to early attached IVF sperm, presumably derived from cytoplasmic streaming forces^54^, before MPn cortical formation (Fig. 2A1–A6). Following oocyte activation and 2^nd^ PB extrusion, the FPn formed at the animal pole before migrating rapidly toward the MPn along presumed assembled sperm astral microtubules (Fig. 2A7–A12 ^55^). FPn migration rates (Table 1) measured at 0.539 ± 0.044 µm min^−1^ (n = 48) compared to IVF migration rates of 0.387 ± 0.056 (n = 5; not significant at p < 0.27). ICSI apposed pronuclei aligned mostly para-tangentially to the adjacent cortex (58%; n = 52) but also obliquely to the A → V axis (86%; n = 52; Table 1). Conversely, ICSI in the oocyte’s central cytoplasm showed decondensing MPn moving by cytoplasmic streaming within the cytoplasm, translocating to the cortex only after FPn apposition (Fig. 2B1–B6). FPn migration rates measured 0.688 ± 0.118 µm min^1^ (Table 1; n = 4), with shorter migration distances. The apposed pronuclei largely aligned para-tangentially to the overlying cortex and obliquely to the A → V axis after migration (75%; n = 4; Table 1).

Taken together, multidimensional TLVM observations of ICSI rhesus zygotes showed extensive cortical spermatozoon displacement and unique para-tangential pronuclear alignment relative to the overlying cortex, but not the A → V axis, compared to IVF-fertilized zygotes. Volumetric changes in the shape of zygotes, along with cytoplasmic streaming forces, might influence the cortical pronuclear para-tangential alignment crucial to cleavage plane development at mitosis.

### The ICSI zygote asymmetric cleavage furrow originates near the cortical male pronuclear breakdown site, progressing though the animal pole, but not the sperm entrance site

In mice, the first cleavage plane may not be random but dependent on being parallel to the line of pronuclear apposition and within 30° of the second polar body and sperm incorporation site^26^. Alternatively, mouse zygotes may cleave perpendicularly to the plane of the approaching male and female pronuclei, irrespective of the 2^nd^ PB or sperm incorporation site, or orthogonally to the bilaterally symmetrical zygote, regardless of the 2^nd^ PB, sperm incorporation site, or pronuclear orientation^27,56,57^.

**Figure 3:**
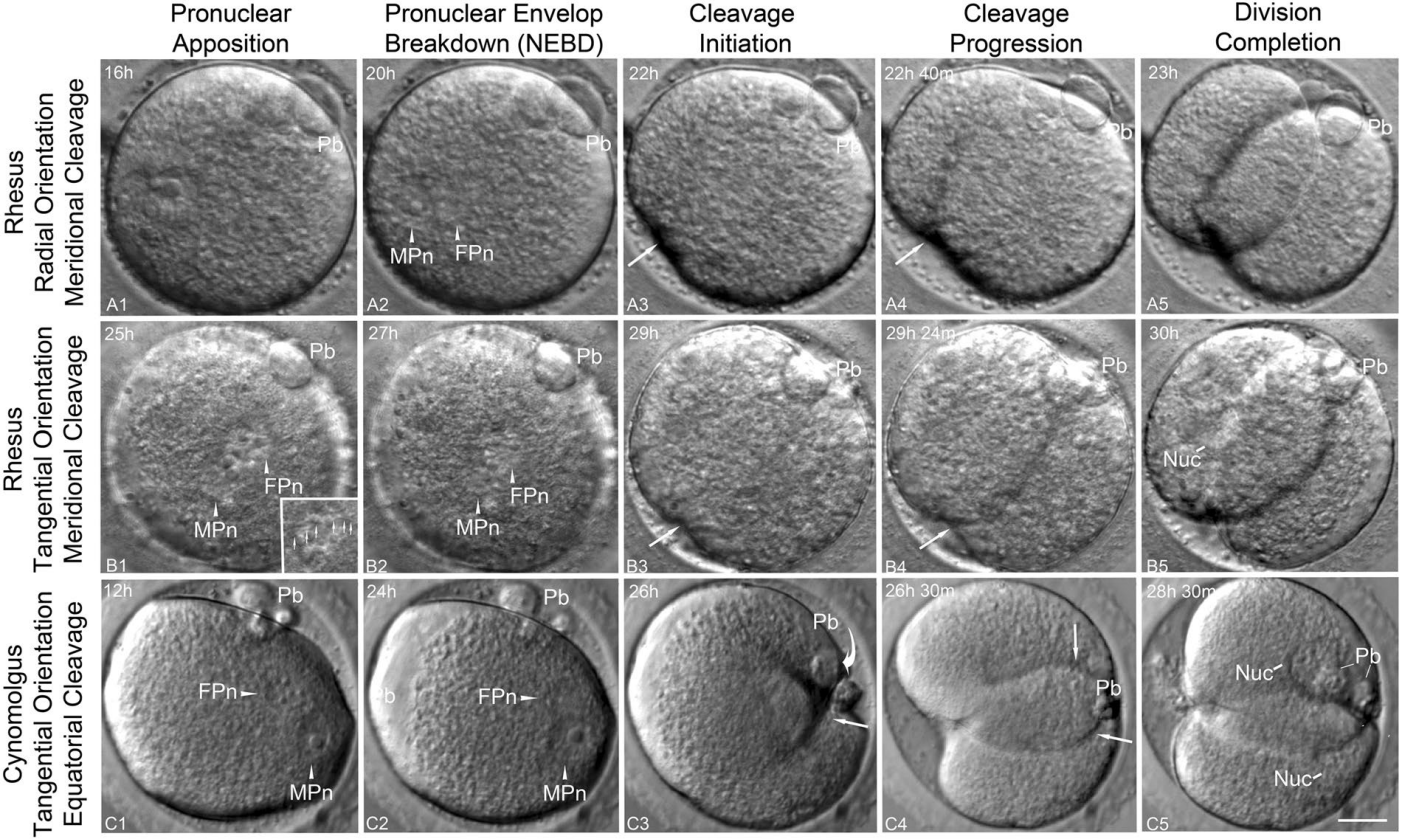
Cleavage furrow initiation and zygotic division in NHP zygotes with varying pronuclear orientations. (**A1**–**A5)** Rhesus zygote showing radially aligned apposed pronuclei with oblique orientation to the A → V axis 16 hrs post-ICSI (**A1**; polar body, Pb). Female pronuclear breakdown occurs first, around 20 hrs post-ICSI (**A2**). A meridional cleavage begins at the cortex overlying the site of the formerly intact MPn (**A3**: arrow), progressing asymmetrically through the cytoplasm to within 30° of the second polar body (Pb) on the opposing cortex (**A4**: arrow) 22 h post-ICSI. After division at 23 hrs post-ICSI, both Pb reside between the daughter blastomeres (**A5**). (**B1**–**B5)** A rhesus zygote with para-tangentially aligned cortical parental genomes oriented along the A → V axis 25 hrs post-ICSI (**B1**; polar body, Pb; inset, details of apposed pronucleus and the incorporated sperm axoneme, small arrows). At 27 hrs post-ICSI, pronuclear breakdown begins with the MPn (**B2**; MPn). Cleavage furrow initiation (**B3**: arrow) begins at the cortex overlying the site of the formerly intact MPn, progressing quickly through a meridional plane that includes the second polar body (**B4**: Pb). Cytokinesis at 30 hrs post-ICSI creates equal-sized daughter blastomeres with the polar bodies residing between the cleaved cells (**B5**: Pb; Nuc: daughter cell nucleus). (**C1**–**C5)** A cynomolgus ICSI zygote at 12 hrs post-ICSI showing apposed parental genomes aligned para-tangentially to the cortex and obliquely to the A → V axis (**C1**). The FPn undergoes breakdown first at 24 hrs post-ICSI (**C2**). However, cleavage furrow initiation at 26 hrs post-ICSI moves in an equatorial direction or right angle to the A → V axis (**C3**, straight arrow) while cortical membrane flow rotates both polar bodies into the developing furrow (**C3**, Pb, curved arrow depicts rotation direction). The bifurcated cleavage furrow progresses towards the opposite cortex (**C4**; arrows) as cytokinesis forms equal-sized daughter blastomeres with the polar bodies between daughter cells by 28.5 hrs post-ICSI (**C5**: polar bodies, Pb; Nuc: reconstituted daughter cell nuclei). All panels are HMC images taken from TLVM videos of ICSI fertilized zygotes undergoing first cleavage. Time in hours:minutes (h:m) post-ICSI. Bar = 20 μm.

We investigated cleavage plane initiation and progression by multidimensional TLVM in ICSI NHP zygotes undergoing first cell division (Fig. 3; Supplemental Videos 4, 5). Apposed radial (Fig. 3A1) or para-tangential (B1, C1) pronuclei began nuclear envelope breakdown (NEBD) 20–27 hrs post-ICSI, beginning with the FPn (36/52; 69%), with the MPn 16–20 min later (Fig. 3A2,B2,C2). Cleavage furrow initiation began cortically, at the cortex closest to where the formerly intact MPn was last observed (Fig. 3A3,B3,C3) and progressing toward the 2^nd^ PB (Fig. 3A4,B4). Cleavage furrow progression nearly always aligned to the animal pole (Table 2), moving diagonally across the zygote and sweeping the polar bodies into the cleavage furrow (Fig. 3C3). After division, equivalent blastomeres formed, with the polar bodies lying between the daughter cells, indicating a meridional cleavage (Fig. 3A5,B5,C5).

**Table 2:** NHP Zygote Cleavage Patterns in Relation to Pronuclear Cortical Orientation and the Zygotic A → V Axis Analyzed from TLVM Recordings.

Insem type	N scored	Sperm MIP and pronuclear orientation to overlying cortex at NEBD	Cleavage initiation at last position of: (%)			Cleavage furrow angle relative to the animal pole [2^nd^ PB] (%)			Cleavage type (%)		2^nd^ PB between daughter blastomeres post division (%)	Blastomere size post division (%)	
			♂PN	♀PN	Other^a^	0–30°	31–60°	>60°	Mer	Eq		Equal	Not Equal
ICSI	19	cortical radial	15/19 (79)	1/19 (5)	3/19 (16)	13/19 (68)	4/19 (21)	2/19 (11)	13/19 (68)	6/19 (32)	16/19 (84)	7/19 (37)	12/19 (63)
ICSI	37	cortical para-tangential	25/37 (68)	0	12/37 (32)	26/37 (70)	5/37 (14)	6/37 (16)	26/37 (70)	11/37 (30)	31/37 (84)	15/37 (41)	22/37 (59)
Partho	5	NA	NA	0	5/5 (100)	3/5 (60)	2/5 (40)	0	3/5 (60)	2/5 (40)	5/5 (100)	4/5 (80)	1/5 (20)

Insem: insemination; Mer: meridional cleavage; Eq: equatorial cleavage; NEBD: pronuclear envelop breakdown; Partho: parthenogenetic chemical activation by ionomycin/6-DMAP exposure; no sperm DNA contribution; NA: not applicable.

^a^includes random cortical and/or 2^nd^ PB sites.

Table 2 summarizes NHP radial versus para-tangential pronuclear alignment with cleavage patterns. Regardless of pronuclear orientation, cleavage overwhelmingly initiated at the cortex adjacent to the last position of the intact MPn, passing within 30° of the animal poles (meridional cleavage), with the 2^nd^ PB lying between daughter cells after division. However, cell division after ICSI did not always result in equal-size daughter blastomeres (37–41%; Table 2), and cytoplasmic fragmentation was notable.

**Figure 4:**
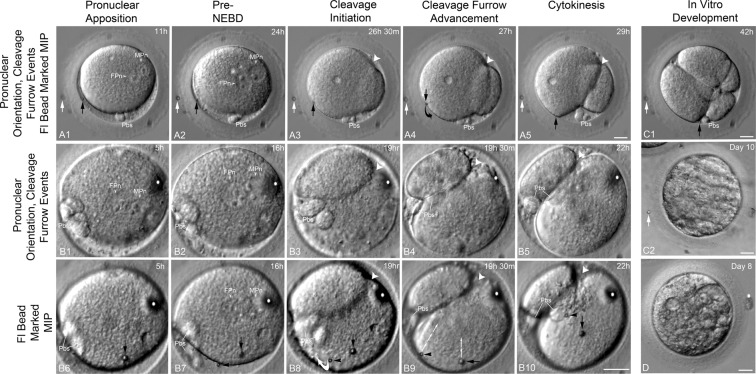
Neither cleavage furrow initiation nor progression includes passage through the sperm microinjection point (MIP). (**A1**–**A5**) A Baboon zygote with radially aligned apposed pronuclei oriented obliquely to the A → V zygotic axis. A detached 3-micron bead within the perivitelline space marks the MIP (**A1**, black arrow). Before mitosis, the expanding cytoplasm re-engages the bead (**A2**, black arrow). An asymmetric cleavage furrow forms near the previously intact apposed pronuclei (**A3**, large arrowhead), progressing meridionally through the polar bodies (Pbs), but not the MIP (**A3**, black arrow). The attached bead rotates by surface membrane flow toward the furrow (**A3**–**A4**, black arrows). Post-cytokinesis, the embryo shows unequal cleavage, with polar bodies in the division plane and the bead near the furrow (**A5**, black arrow). (**B1**–**B10**). A Baboon zygote showing pronuclear events (**B1**–**B5**) and movement of two attached marking beads during cytokinesis (**B6–B10**). A 5-micron bead marks the MIP (**B6**, black arrow); the 3-micron bead marks a distal cortex site (**B7**, black arrowhead). Radially aligned apposed pronuclei are oblique to the A → V axis (**B1**) with a bead-marked MIP (**B6**: black arrow). The apposed pronuclei translocate cortically, aligning with the A → V axis pre-mitosis (**B2**). Slight membrane flow exposes both attached beads (**B7**, black arrow, arrowhead). Furrow initiation occurs near the formerly intact MPn (**B3**, white arrowhead), proceeding meridionally toward the polar bodies (Pbs), but not through the beads (**B8**, black arrow, arrowhead). Beads show independent movement; the 3-micron bead initially toward the polar bodies (**B8**, white curved arrow) before redirecting toward the furrow, while the 5-micron MIP bead moves vertically toward the mid-furrow (**B9**, small white arrows). Cytokinesis is unequal, with polar bodies (Pbs) between blastomeres and both beads near the furrow (**B10**, black arrow, arrowhead). (**C1**–**C2**). The **A1–A5** Baboon zygote after *in vitro* development. Second division is equatorial in both daughter cells, with the MIP bead near the polar body between blastomeres (**C1**; black arrow). (**D**) The **B1–B10** baboon blastocyst on Day 8 post-*in vitro* culture. All panels are HMC images. Time (hours:minutes) post-ICSI. Vac: cytoplasmic vacuole(s). White arrow: zona pellucida reference bead; White ^*^zona pellucida surface debris. Bars = 20 μm.

Investigations on cleavage initiation and progression in relation to marked ICSI microinjection points (MIPs) using a phytohemagglutinin-labeled 3- to 5-µm fluorescent bead is shown in Fig. 4 and Table 3. In most cases, whether apposed pronuclei were radially or para-tangentially oriented, the cleavage furrow neither initiated at, nor progressed through, the MIP (Table 3). The fluorescent beads marking the MIP site moved toward cleavage furrows owing to cortical rotation (Fig. 4A1–A5,B1–B10; Supplemental Videos 6, 7). Cleavage was consistently unequal after first division, although *in vitro* development to morula or early blastocyst stages appeared uncompromised (Fig. 3C1,C2,D).

**Table 3:** NHP Zygotes Do Not Initiate or Progress the Cleavage Furrow through the ICSI Microinjection Point (MIP).

Sperm MIP/Cortical Pronuclear Orientation to A → V axis	Total Zygotes Scored	Cleavage Furrow Initiation and Progress in relation to the MIP site marked by a Fluorescent Bead			
		initiation at the MIP (%)	progression through the MIP (%)	both initiation and progression through the MIP (%)	Neither initiation or progression through the MIP (%)
Cortical/radial	7	0	1/7 (14)	1/7 (14)	5/7 (71)
Cortical/para-tangential	7	0	2/7 (29)	1/7 (14)	4/7 (57)

Collectively, first division in ICSI zygotes begins asymmetrically at the cortex nearest where the intact MPn was last observed and not the sperm injection site. Cleavage progression is mostly toward the animal pole (2^nd^ PB) in a meridional pattern. Cortical rotation translocates both the polar bodies and the MIP into the furrow region, consistent with a previous report describing the sperm injection site as localized near the first cleavage plane after cell division^30^.

### Spindle orientation and microtubule:cortex interactions during division differ between IVF- and ICSI-fertilized zygotes

In mice, acentriolar maternal microtubule organizing centers (MTOCs) nucleate cytoplasmic microtubules that “direct” pronuclear apposition to the oocyte’s center, forming the centralized acentriolar, anastral, barrel-shaped bipolar mitotic spindles (Supplemental Fig. S2 ^12^). Mitotic chromosome segregation is concomitant with an elongating spindle apparatus, crisscrossing opposite-pole microtubules, and a symmetrical invaginating furrow mediated by myosin II, the microfilament-associated motor protein involved in cleavage furrow activity^58^.

**Figure 5:**
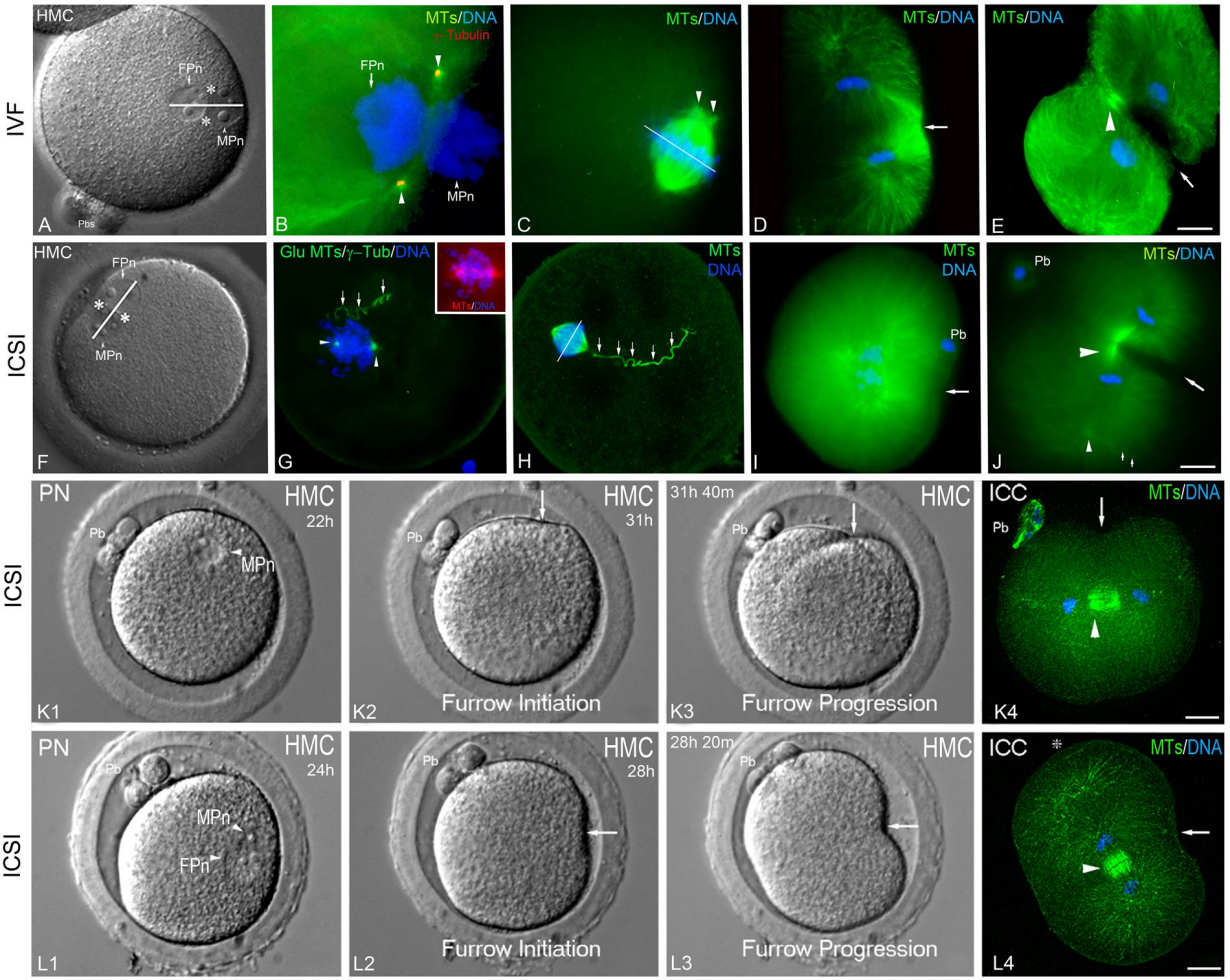
Mitotic spindle assembly and first cleavage patterns differ between IVF- and ICSI-Fertilized NHP Zygotes. Rhesus IVF- (**A**–**E**) or ICSI- (**F**–**J**) fertilized zygotes. (**A**,**F**) Interphase zygotes with radially oriented (IVF: **A**) versus para-tangentially (ICSI: **F**) aligned apposed male and female pronuclei. White asterisks: duplicated centrosome positions; white bars: spindle equator chromosome alignment and anaphase cleavage plane direction. (**B**,**G**) fixed immunostained zygotes with duplicated, split centrosomes (arrowheads) juxtaposed between the apposed male and female pronuclei (blue) and nucleating astral microtubules (green; (**G**) inset, total tubulin [red] and DNA [blue]; incorporated sperm axoneme [small arrows]). (**C**,**H**) fixed metaphase spindles immunostained for microtubules (green) and DNA (blue), showing eccentric and tangentially-oriented IVF spindles, are distinct from radially oriented, cortically distal ICSI spindles (**H**: sperm axoneme, small arrows). White bars: the orthogonal anaphase chromosome separation plane. (**D**,**I**) A fixed, immunostained IVF anaphase spindle with enhanced spindle microtubules (**D**: green) signaling cortical cleavage furrow initiation (arrow) compared to an ICSI anaphase spindle lacking cortical microtubule interaction at the furrow (**I**: green, arrow). Both cleavage planes are orthogonal to the separating sister chromosomes (blue). (**E**,**J**) Equal IVF telophase (**E**) cleavage versus unequal ICSI telophase cleavage (**J**) despite asymmetric cleavage furrows (arrows) progressing orthogonally to midbodies (green, arrowheads; blue, DNA; small arrowhead: centrosome; small arrows: sperm axoneme). (**K1-K4**, **L1-L4**). TLVM images of two ICSI rhesus zygotes, both with eccentric, radially aligned, apposed male and female pronuclei (**K1**, **L1**). The cleavage furrow begins near the previously intact MPn (**K2**, **L2**: arrows), progressing asymmetrically (**K3**, **L3**: arrows) but not through the second polar bodies (Pb). (**K4**,**L4**) anti-tubulin (green) and Hoechst DNA (blue) immunostaining of (**K3**,**L3)** zygotes fixed 2 min post-live imaging. Central microtubule midbodies (green, arrowheads) lie orthogonal to the progressing cleavage furrows (arrows). Polar bodies are excluded from the cleavage planes (**K4**: Pb; **L4**: * marks lost polar bodies). HMC images: (**A**,**F**,**K1**–**K3**,**L1**–**L3)**; **B**: triple-labeled for microtubules (green), γ-tubulin (red), and DNA (blue); (**G**) quadruple-labeled for glutamylated-microtubules/γ-tubulin (green), total tubulin (red), and DNA (blue); (**K4**,**L4**) microtubules (green) and DNA (blue). Pb: polar bodies. Bars = 20 μm.

Unlike mice, other mammals, including humans and NHPs, inherit the dominant MTOC from the sperm (the paternal centrosome) to assemble the anastral bipolar mitotic metaphase spindle (Fig. 5 ^12,52^). The paternal centrioles in most mammals include the resurrection of a highly atypical distal centriole that undergoes duplication during interphase to provide the basis for mitotic bipolar spindle assembly^14,15^. The proximal sperm centriole remains with the incorporated sperm axoneme found on the paternal pronucleus, and by late interphase, the now duplicated paternal centrioles locate at the leading edges of the overlapping apposed pronuclei^52^. The mitotic spindle has one pole that retains the proximal centriole attached to the sperm tail and one pole with the resurrected distal centriole^14^.

In NHP zygotes, pronuclear orientation impacts cytoplasmic bipolar spindle orientation after NEBD (Fig. 5; Supplemental Fig. S3). For IVF-fertilized zygotes at late interphase, radially oriented pronuclei tightly associate with the adjacent cortex, and the centrioles mark the poles of assembled para-tangential spindles (Fig. 5A–C). Male and female chromosomes condense separately, align at the metaphase spindle equator, and division proceeds orthogonally to chromosome separation (Fig. 5C). At anaphase, spindle microtubules form transient overlapping bundles at the adjacent cortex, signaling asymmetric cleavage furrow formation (Fig. 5D, arrow; 5/5 IVF anaphase spindles). Midbody microtubules anchor the spindle apparatus to the invaginating furrow as it moves toward the opposing cortex (Fig. 5E). This pattern of spindle assembly and cleavage initiation favors equal daughter cell formation after cytokinesis (15/23; 65%).

ICSI zygotes have more random pronuclear orientations (Table 1). Para-tangential pronuclear orientation positions centrioles favoring radially aligned mitotic spindles relative to the adjacent cortex (Fig. 5F–J; Supplemental Fig. S3). Observationally, assembled ICSI mitotic spindles appear at greater distances from the overlying cortex, with reduced or absent microtubule:cortex interactions (Fig. 5I,K1–K4,L1-L4; 2/14;14%) that may cause cell division errors. However, ICSI zygotes form asymmetric cleavage furrows that pass orthogonally to segregating chromatids at anaphase but with more unequal cleavage divisions (Table 2; 25/42; 60%).

### Cytoplasmic pronuclear orientation, cleavage patterns, and microtubule configurations in parthenogenotes and androgenotes demonstrate that cortical affinity lies with the male pronucleus

Understanding of male and female pronuclear orientation benefits from studying parthenogenotes (no male contribution) and androgenotes (male contribution only). Parthenogenotes, created either by chemical treatment with ionomycin/6-DMAP or by injecting metaphase-II arrested oocytes with a crude sperm extract, form haploid or diploid female pronuclei that migrate randomly within the central cytoplasm (Supplemental Fig. S4). Conversely, androgenotes, created by injection of a single sperm into an “enucleated” oocyte (meiotic spindle:chromosome complex removed by a micropipette), form a single, cortex-associated MPn (Supplemental Fig. S4). A strong correlation exists between a decondensed MPn and eccentric cortical pronuclear positioning in NHP oocytes, although how this is initiated and maintained is not currently known.

TLVM recordings of parthenogenote and androgenote zygotes undergoing first mitosis are shown in Supplemental Fig. S5 and Supplemental Video 8. For parthenogenotes, mitotic spindle assembly occurred within the central cytoplasm after FPn breakdown, with an asymmetric cleavage furrow assembling from either the animal pole or a random cortical site (Table 2). Androgenotes, with the MPn closely associated with the overlying cortex, assembled cortical spindles and asymmetric cleavage furrows at the adjacent overlying cortex as observed for ICSI-fertilized zygotes (Supplemental Fig. S5; Supplemental Video 8).

Microtubule and DNA patterns in mitotic parthenogenotes are presented in Supplemental Fig. S6. Identical microtubule and DNA configurations were observed regardless of the artificial activation methodology employed. Cytoplasmic microtubule patterns in late interphase parthenogenotes were random in the absence of sperm centrioles. At mitosis, a bipolar anastral central cytoplasmic spindle assembled with aligned female chromosomes, probably directed by specific microtubule-based molecular motor proteins that participate in non-centrosomal spindle assembly^59^. By anaphase, spindle microtubules were sparse in the equatorial region through which cleavage furrow progression would ensue, and despite asymmetrical cleavage furrow formation and progression, no spindle microtubule:cortex interactions were observed (n = 6).

Collectively, a strong cortical attraction of the MPn to the zygote’s oolemma directs cleavage furrow formation as mitosis ensues. Absent any paternal contribution, female pronuclei lack dominant MTOCs (centrioles) to direct motility events within the activated cytoplasm, including mitotic spindle assembly. Parthenogenotes demonstrate lower affinity for cortical pronuclear positioning, central cytoplasmic mitotic spindles with enormous spindle pole microtubule assembly, but no anaphase microtubule:cortex interactions despite asymmetric furrows that pass orthogonally to the chromosome separation plane. The molecular signals required to initiate cleavage furrow assembly and progression in parthenogenotes are currently not known.

## Discussion

Notwithstanding excellent investigations conducted on the best-studied mammalian model, the mouse, crucial information is scarce regarding early fertilization events influencing axis formation in non-rodent mammals^30,60–62^. Controversy surrounds the evidence indicating that developmental information encoded within oocytes and early zygotes determines axis development of the blastocyst; strengths and limitations of the various experimental designs have been discussed^17^. For ART clinics, whether understanding of early morphological characteristics in the human zygote can translate to selection of the highest quality human embryos is enormously important ^36^. Animal models like NHPs provide more translatable relevance to humans^13,63^. For example, atypical early fertilization events after ICSI, such as abnormal nuclear remodeling, asynchronous apical sperm chromatin decondensation, and inappropriate retention of sperm proteins during interphase, have been identified in NHP zygotes^64,65^, which may have important implications for ART, considering the growing use of ICSI for oocyte insemination.

Here, observational information in macaques and baboons, using continuous live multidimensional (z-stack) TLVM recordings, from sperm incorporation though first division, is provided, a crucial improvement over single-plane imaging or only fixed-stage microscopic examinations. Multidimensional imaging permits observing independent male and female pronuclear behaviors, pronuclear migration rates and ultimate oolemma orientation and cortical marking sites (i.e., polar bodies and sperm entrance sites) as well as cleavage furrow initiation, progression, and cytokinesis in 4D (3D + time). NHP oocytes and zygotes, as large ovoid spheres, benefit from 4D imaging, where detailed motility analysis on the entire fertilization process can be studied in detail to identify robust embryos. Complementing multidimensional TLVM analysis was the fixation of mitotic zygotes in the investigation of spindle arrangements and cleavage events as influenced by pronuclear orientation, including analysis of the same zygote undergoing TLVM imaging within minutes of the last captured frame (Fig. 5). Additionally, manipulating the genetic constitution of zygotes by forming parthenogenotes and androgenotes is informative regarding the interactions of male and female pronuclei within the activated zygote’s cytoplasm. These observational studies lay a foundation for studying the underlying molecular mechanisms driving asymmetric pronuclear apposition, spindle assembly, and cleavage initiation in a higher-order primate model with better potential extrapolations to humans than studies in mice.

This study identified distinct differences in sperm head behavior between NHP IVF- versus ICSI-inseminated zygotes (Figs 1 and 2). IVF sperm appeared tightly bound to the oolemma following membrane penetration, with sperm head movement mediated by increased bulk membrane flow during second meiosis completion (Fig. 1; Suppl Video 1). Typically, IVF sperm heads decondensed and remained closely associated with the cortical penetration site (Suppl Video 2). In contrast, ICSI fertilization showed different dynamics following sperm microinjection (Fig. 2). Placement of the immobilized sperm into the oocyte’s cortex induced sperm head displacement along the inner plasma membrane, probably from cytoplasmic streaming forces (Suppl Video 3). Rarely did cortical ICSI sperm remain at the microinjection site but always showed a strong cortical retention.

The significance of these observations relates to the eccentric orientation of the male and female pronuclei after sperm head decondensation, pronuclear assembly, and migration (Table 1). NHP zygotes have highly eccentric, cortically positioned, juxtaposed male and female pronuclei post apposition, with IVF zygotes showing radial pronuclei orientations relative to the overlying adjacent cortex while ICSI zygotes vary their pronuclear alignment between radial and para-tangential alignments (Table 1; Figs 1 and 2). Furthermore, TLVM analysis showed many ICSI zygotes with atypical pronuclear cytoplasm movements after apposition, including pronuclear tumbling, separation/ejection, and slow drifting away from the cortex prior to mitosis, reinforcing how the lack of sperm:oolemma binding might affect the final ICSI pronuclear cytoplasm positioning that influences cleavage initiation and progression.

Interestingly, while NHP zygotes closely mimic the early fertilization events observed in human zygotes^63^, NHP pronuclear positioning post-apposition is not equivalent to humans, that show more central cytoplasmic apposition post female pronuclear migration^37–49^, like that observed in the mouse^57^. Past studies in human zygotes have shown that pronuclear morphology (nucleoli) and, perhaps, orientation to the polar body impact embryo quality, blastocyst development, and pregnancy establishment, irrespective of fertilization method^41,42^. A recent TLVM analysis of human ICSI zygotes, however, reported nearly half to have cortically or intermediately positioned cytoplasmic apposed male and female pronuclei^41^. It would be interesting to know how human ICSI pronuclear cytoplasmic position and alignment influences cleavage events and translates to human pregnancy outcomes. Taken together, these data indicate how early NHP fertilization events vary between conventional IVF and ICSI insemination, with potentially important implications for clinical ART outcomes.

Neither NHP IVF or ICSI sperm induced a post-insemination incorporation cone (Figs 1 and 2; Suppl Videos 1 and 3), a structure in mouse oocytes that supports first cleavage plane assembly by preferentially aligning the mitotic spindle along the SEP^26,33^. Interestingly, human zygotes reportedly assemble transient insemination cones a few hours after IVF or ICSI insemination, although the frequency of occurrence appears very low^40,41^, while in mice, the site of sperm entry and the 2^nd^ PB axis appear to be important determinants for axial development of the mouse blastocyst^28,29^.

A previous study on ICSI-fertilized rhesus oocytes tagged at the SEP with a fluorescent bead suggested a strong influence of the NHP SEP on early axis specification; this was based on observing embryos at the 2-cell and blastocyst stages after *in vitro* culture^30^. However, continuous multidimensional TLVM in baboon zygotes showed most fluorescent beads tagging the MIP ended up within the furrow region at the end of cytokinesis, resulting from bulk membrane flow directed toward the first division plane as opposed to either cleavage initiation or progression through this site (Fig. 4; Table 3; Suppl Video 6). Attempts to trace where the MIP site resides in the expanded baboon blastocysts grown *in vitro* was confounded by multidimensional TLVM observations showing bead detachments/reattachments during preimplantation development, with overall *in vitro* development rates to the blastocyst stage at 27% (3/11]; Fig. 4). While the MIP did not appear to influence cleavage plane initiation or progression, cleavage furrows overwhelmingly began at the cortical site near where the previously intact MPn resided and progressing meridionally through the animal pole (2^nd^ PB), regardless of whether the ICSI apposed pronuclei were oriented radially or para-tangentially to the overlying cortex (Fig. 3; Table 2). In this regard, Hiiragi and Solter’s^34^ observation that the mouse cleavage plane was anticipated by the plane of the approaching male and female pronuclei appears to explain NHP cleavage initiation also. Tracing a line along the long axis of the NHP apposed pronuclei appears to be an accurate predictor of cleavage plane progression.

For non-rodent mammals, the influence of the animal pole and the SEP shows varying influence on division planes. Human IVF zygotes assemble central cytoplasm mitotic spindles with furrow invagination beginning equally from opposite cortical regions and passing close to the 2^nd^ PB and through the SEP, perhaps indicating a role for the SEP in human axis specification^38,41^, while the SEP in IVF sheep zygotes does not appear to influence the first cleavage plane^60^.

Mouse oocyte shape changes, induced either artificially by external mechanical force or by the sperm incorporation cone at insemination, influence the first cleavage plane assembly by aligning the spindle to the SEP^26,33^. In the early NHP ICSI zygote, multidimensional TLVM showed significant cortical contraction/expansion cycles, sometimes visualized as pulsating images on the TLVM recordings. The appearance of contractions coincided with microtubule aster assembly from the base of the incorporated sperm, arising from sperm astral microtubule interaction with the cortical membrane. Cytoplasmic expansion generally occurred as mitosis ensued, with a net outflow of cytoplasmic constituents away from the assembling first mitotic spindle.

Our TLVM recordings also occasionally detected a “cytoplasmic wave” emanating from the growing sperm aster after ICSI, as described during early human fertilization^41^. However, NHP cleavage plane assembly and progression appears to be more related to the actual male pronuclear site, pronuclear orientation, and the inherited sperm centrosome cytoplasmic positioning by the end of interphase. While both IVF and ICSI NHP sperm contribute centrioles that assemble the zygotic centrosome that nucleates sperm astral microtubules for female pronuclear migration^52^, no statistical differences between IVF and ICSI zygotes’ female pronuclear migration rates were identified (Table 1), although less robust ICSI sperm:cortical interaction seemed to shorten migration distances between distal pronuclei. The NHP reconstituted centrosome, after fertilization, duplicates during interphase^14^, lying between the opposing pronuclear membranes (Fig. 5 ^52^) and defining the first mitotic spindle poles as they do for most mammals except mice^12^. Mice do not assemble a microtubule-based sperm aster with pronuclear movements directed by cortical interphase microtubule arrays and microfilaments^12,66–69^, and the maternal centrosomes that coalesce about the nuclear surfaces of concentric male and female pronuclei prior to mitosis direct spindle assembly (Supplemental Fig. S2 ^68^). However, how pronuclear orientation differed between NHP IVF and ICSI zygotes at the end of interphase appeared to markedly influence eccentric mitotic spindle assembly and cytoplasmic orientation (Fig. 5).

In IVF zygotes, radially oriented apposed male and female pronuclei, with the MPn closest to the overlying adjacent cortex (Fig. 1; Table 1), favors assembling eccentric para-tangential bipolar mitotic spindles, which initiate division planes that move across the greater diameter of the zygote, forming two cells with equal daughter blastomeres (Fig. 5; Suppl Fig. S3). Conversely, ICSI mitotic zygotes show para-tangential pronuclear orientations that have centrosome positions shifted 90° relative to IVF mitotic spindles (Fig. 5). At anaphase, ICSI spindles show reduced spindle microtubule:cortex interactions (compare Fig. 5D,I,K4,L4; 14%), which might critically impact not only furrow initiation signaling but mitotic spindle stabilization during division, so that the rapidly invaginating furrow does not sweep aside the spindle apparatus during cytokinesis to form abnormal binucleate daughters. Mitotic spindle re-orientation within the cytoplasm may be impacted by cortical streaming forces acting on the less well-anchored ICSI spindle, which could account for the greater incidence of unequal sized daughter blastomeres observed post-cytokinesis (Table 2).

The significance of the MPn in cleavage initiation was reinforced by our observations on (i) placement of the microinjected sperm into the central cytoplasm of the oocyte; (ii) cytoplasmic pronuclear location and cleavage in parthenogenetic zygotes; and (iii) pronuclear location and cleavage in androgenetic oocytes. For sperm central cytoplasmic deposit, the female migrated to the male quickly (Table 1) but once apposed, moved precipitately toward the cortex in tandem (Fig. 2), with cleavage beginning at the cortex closest to the previously intact MPn, usually identified by the attached incorporated sperm axoneme. In parthenogenotes, formed by either chemical activation (ionomycin/6-DMAP) or by microinjection of a crude sperm extract, the female pronuclei generally moved toward the central cytoplasm, away from the cortex (Supplemental Fig. S4) and assembled asymmetric cleavage furrows, mostly from the polar body region, but sometimes, from random cortical sites (Table 2; Supplemental Fig. S5).

Investigations on fixed mitotic parthenogenotes showed the random cortical microtubule arrays formed after oocyte activation in the absence of male-contributed centrioles as well as cytoplasmic centric anastral, acentriolar bipolar spindles at metaphase (Supplemental Fig. S6). Interestingly, mitotic chromosome separation at anaphase was concomitant with large spindle pole microtubule assembly that appeared to be excluded from any cortical region. Cleavage progression, however, was orthogonal to the separating chromosomes. These NHP parthenogenote observations support findings in mouse parthenogenotes, where, in the absence of an SEP, cleavage planes were observed to be random^70^.

Lastly, we investigated pronuclear events and cleavage initiation in androgenotes produced either by meiotic spindle enucleation followed by ICSI or removal of the FPn shortly after ICSI and FPn formation. Regardless of genetic constitution, enucleated oocytes had male pronuclei with a strong cortical attraction (Supplemental Fig. S4) and cleavage initiation at the cortex closest to the previously intact MPn as observed for ICSI-fertilized zygotes (Supplemental Fig. S5). We speculate that sperm centrosomal microtubules, interacting with unknown cortical proteins, drive male pronuclear cortical positioning, although the exact mechanism remains unknown. Collectively, these observations show a strong attraction of the paternal pronucleus for the cortex and eccentric cleavage initiation, which may reflect the assembly of the sperm microtubule aster from the reconstituted centrosomes following insemination.

In summary, NHP zygotes display features distinct from more traditionally studied rodent models, with fundamental differences in pronuclear cytoplasmic positioning/orientation, spindle assembly in relation to inherited centrosome placement, and cleavage initiation/progression. ICSI cleavage initiation and progression is independent of a SEP but favors division through the animal pole. Nonhuman primates follow the pattern of paternal inheritance of centrosomes for directing motility events during fertilization seen also in humans, but not mice^12^. Differences between NHP and mouse zygotes are profound in many aspects of the fertilization process, including sperm incorporation mechanics, paternal centriole inheritance, male and female pronuclear apposition methods, atypical cytoplasmic position of juxtaposed pronuclei, mitotic bipolar spindle assembly mechanisms, and cytokinesis characteristics, among others (Supplemental Table 1). Identifying these unique characteristics has helped to define how NHP zygotes accomplish zygotic division as the first step in the process of embryonic axis formation and sets the stage for investigation of the molecular signals directing these critical events.

## Methods

### Ethics statement

All research protocols complied with the National Institute of Health’s Office of Laboratory Animal Welfare *Guide for the Care and Use of Laboratory Animals* regulations under the full approval of protocols by the Institutional Animal Care and Use Committees (IACUCs) of Oregon National Primate Research Center (NOI #s 0483; 0095), Magee-Womens Research Institute & Foundation at the University of Pittsburgh (IACUC #s 0802725, 0804730), and the Texas Biomedical Research Institute in San Antonio Texas (formerly known as the Southwest Foundation for Biomedical Research; IACUC#s 1235 MF, 1168 MF, 986 PC 21, and 1235 MF).

### Non-human primate gamete collections

Ovarian stimulation of female rhesus monkeys exhibiting regular menstrual cycles was induced with exogenous gonadotrophin administration as previously described^71^. Follicular aspirations were performed approximately 27–30 h post-hCG via laparoscopy for macaques (rhesus, cynomolgus) and around 32–36 h post-hCG for the baboon^52,71^. Oocytes at metaphase II-arrest, exhibiting expanded cumulus cells, a distinct perivitelline space, and first polar body, were selected for IVF fertilization, artificial activation, or ICSI^55,64,72^.

### Fertilization, artificial activation, and preparing androgenetic NHP oocytes

Rhesus and cynomolgus sperm were collected by penile band electroejaculation and fertilized *in vitro* as reported^73^. Baboon sperm was collected by rectal probe electroejaculation under approved protocols^71^. ICSI fertilization using 6–7 μm microinjection needles (Humagen, Inc., Charlottesville, VA) was accomplished by the methods of Hewitson *et al*.^55^. In some cases, a small (~3 µm) fluorescent bead (Polysciences, Inc, Warrington, PA) coated with 350 µg/ml phytohemagglutinin (PHA; Sigma, St. Louis, MO) was placed onto the membrane to mark the ICSI site after sperm delivery, as described by Piotrowska and Zernicka-Goetz^70^. Artificial activation (parthenogenesis) was accomplished by either activation with 5 μm ionomycin/1.9 mM 6-dimethylaminopyridine (6-DMAP)^74^ or injection of an NHP crude sperm extract (120–240 pg ml^−1^^75^). Androgenote production was accomplished by ICSI of oocytes after enucleation of the second meiotic spindle or the female pronucleus 4–6 hrs post ICSI using a 22-µm pipet as previously reported^76,77^.

### Immunocytochemistry

Selected zygotes were fixed either after zona pellucida removal overnight in 2% formaldehyde or for 15 mins in absolute methanol (−20 °C) following extraction in a detergent based stabilization buffer^78^. Primary antibodies (1 hr; 37 °C) included anti-tubulin (E-7; 1:20; Developmental Studies Hybridoma Bank, Iowa City, IA), γ-tubulin (1:500; gift of Dr. T. Stearns, Stanford University), myosin II (gift of Dr. P de Lanerolle, University of Illinois-Chicago); pericentrin (PCM; gift of Dr. S. Doxsey, University of Massachusetts Medical School); and detyrosinated anti-tubulin (glu-MTs; 1:500; Millipore, Temecula, CA). Primary antibodies were detected with appropriate fluorescein- or rhodamine-labeled secondary antibodies (1:200; Molecular Probes, Eugene, OR.). DNA was labeled with 10 µg/ml Hoechst 33345 (Sigma Chemical Corporation, St Louis, MO). Images were taken by conventional epifluorescence or laser-scanning confocal microscopy (Leica LCS, Leica Microsystems Heidelberg, Germany).

### Time-lapse video microscopy

(TLVM) was performed on an inverted Nikon TE-2000U using Hoffman Modulation Contrast (HMC) optics (x 20). An environmental chamber equipped with a heater to maintained 37 °C encased the microscope (Fryer Environmental Control Chamber, Chicago, IL). A 35 mm glass bottom Petri dish (MatTek, Ashland MA) was placed into a Tokai Stage Top Chamber Unit (Model: INU-ONI-F1; Tokai Hit, Japan) mounted to the microscope stage to maintain pH by a constant flow of 5% CO_2_ gas. Images were collected using an ORCA-ER digital camera (Hamamatsu Photonics, Inc., Japan) using Metamorph software (Molecular Devices, Sunnyvale, CA). Z-series control for acquisition of image stacks was accomplished with a z-motor (Model MFC-2000; Applied Scientific Instruments, Eugene, OR) and controlled by Metamorph. Z-series images (5 µm) of pronuclear development, apposition and migration were collected every 5–15 mins while mitosis was collected every 2 min.

### Imaging and analysis

Conventional fluorescence microscopy was performed using a Nikon Eclipse E1000 microscope with high numerical aperture objectives and data recorded digitally using a cooled CCD camera (Hamamatsu Instruments Inc., Japan) and Metamorph software (Universal Imaging, West Chester, PA). Laser-scanning confocal microscopy utilized a Leica SP-2 equipped with argon and helium–neon lasers. Selected image panels from TLVM recordings were prepared with time listed in hrs:min. For all images, final panels for plates were prepared from selected tagged imaged file format (tiff) images in Adobe Photoshop (Adobe Systems, Inc, San Jose, CA).

### Statistics

Means ± standard error (s.e.) were determined by an online tool at EasyCalculations.com. Statistical significance was determined with the Student’s *t* test (two-tailed), with actual p valves expressed, and was performed with GraphPad Software, Incorporated (La Jolla, CA). Significance was determined if p < 0.05.

## Supplementary information

Supplementary Data

Supplemental Video 1

Supplemental Video 2

Supplemental Video 3

Supplemental Video 4

Supplemental Video 5

Supplemental Video 6

Supplemental Video 7

Supplemental Video 8

## Data Availability

Datasets generated and/or analyzed during this study are available from the corresponding author on reasonable request as set forth in the journal guidelines.
