# Supplementary material for: Fertilization and Cleavage Axes Differ In Primates Conceived By Conventional (IVF) Versus Intracytoplasmic Sperm Injection (ICSI)
Source: Sci Rep. 2019 Oct 25;9:15282. doi: 10.1038/s41598-019-51815-4 (PMC6814755; doi:10.1038/s41598-019-51815-4)
Supplement: Supplementary file 1 — Supplementary Data [file 41598_2019_51815_MOESM1_ESM.pdf]

**SUPPLEMENTARY DATA FOR**

# FERTILIZATION AND CLEAVAGE AXES DIFFER IN PRIMATES CONCEIVED BY CONVENTIONAL (IVF) VERSUS INTRACYTOPLASMIC SPERM INJECTION (ICSI)

by

Calvin R. Simerly[1], Diane Takahashi[2], Ethan Jacoby[3], Carlos Castro[1], Carrie Hartnett[1], Laura Hewitson[4], Christopher Navara[5] & Gerald Schatten[1,*]

[1] Magee Womens Research Institute; Pittsburgh Development Center, Division of Developmental & Regenerative Medicine, and Departments of Obstetrics-Gynecology-Reproductive Sciences, Cell Biology and Bioengineering; University of Pittsburgh Cancer Institute, University of Pittsburgh School of Medicine, 204 Craft Avenue Pittsburgh, Pennsylvania 15213, USA

[2] Division of Diabetes, Obesity & Metabolism, Oregon National Primate Research Center, Oregon Health & Science University, Beaverton, Oregon 97006, USA

[3] CCRM Houston Main Center Memorial City, 929 Gessner Rd, Suite 2300, Houston, Texas 77024, USA

[4] The Johnson Center for Child Health and Development, Austin, Texas 78701, USA

[5] Department of Biology, South Texas Center for Emerging Infectious Disease, University of Texas at San Antonio, San Antonio, Texas 78249, USA

*Correspondence: (412) 641-2403 (phone); (412) 641-6342 (fax)

Email: schattengp@upmc.edu

**SUPPLEMENTAL FIGURES.**

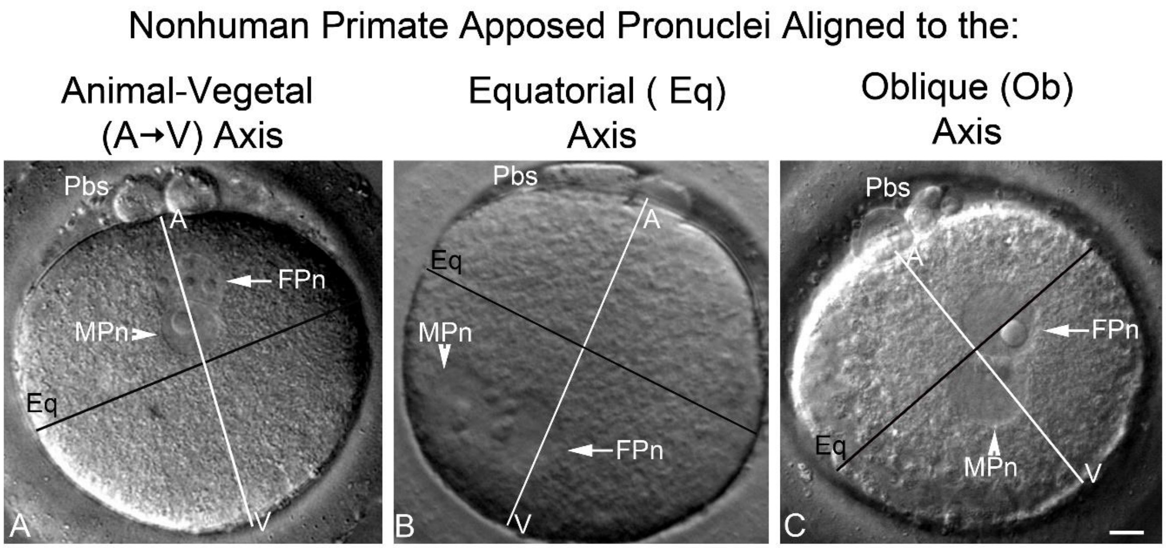


**Supplemental Figure S1. Cytoplasmic Orientation of Apposed Male and Female Pronuclei Relative to the Animal-Vegetal (A→V) Axis in NHP Zygotes.** By convention, the 2nd polar body defines the animal pole in mammalian oocytes. Here, the white lines depict the animal-vegetal axis of the NHP zygote, while black lines show the equatorial axis. A: Apposed male and female pronuclei aligned along the A→V axis (white line) prior to the onset of pronuclear envelope breakdown. B: Eccentric apposed male and female pronuclei aligned along the equatorial axis (black line) in a fertilized zygote**.** C: Apposed cortical male and female pronuclei aligned obliquely to either the A→V or equatorial axis. Most NHP zygotes with apposed pronuclei align oblique to the A→V axis. Bar= 10μm.

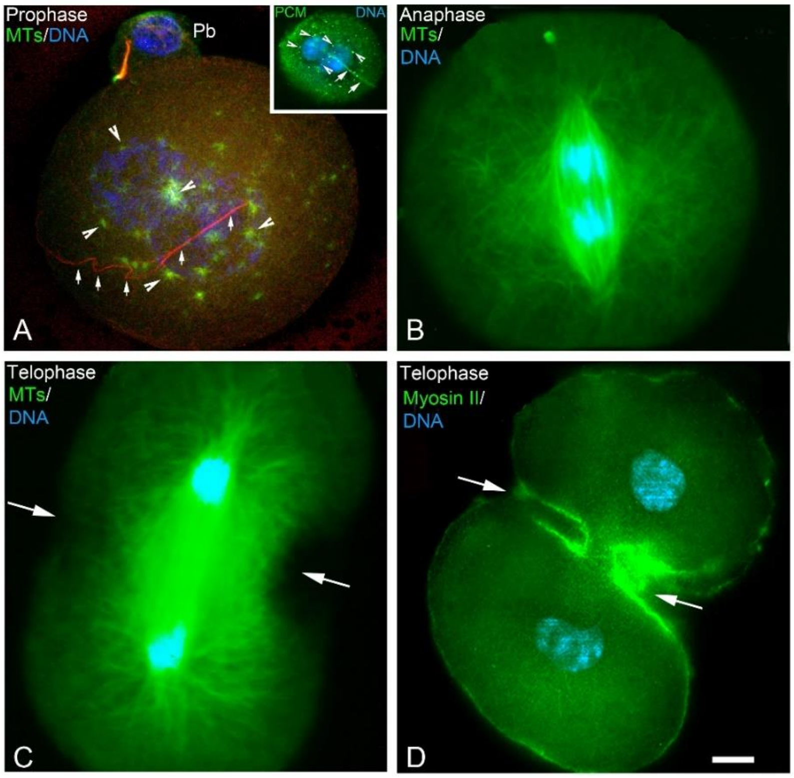


**Supplemental Figure S2. Microtubules and Myosin II during Cleavage of Fertilized Mouse Zygotes.** **A**: Prophase. The apposed male and female pronuclei beginning nuclear condensation (blue) are surrounded by numerous small microtubule asters on both pronuclear surfaces (green, arrowheads). The incorporated sperm axoneme lies in the cytoplasm (red, small arrows), and the meiotic midbody connects the ejected second polar body (Pb) to the zygote. Inset: The acentriolar maternal pericentriolar material that nucleates cytoplasmic microtubules are shown aligned along the male and female pronuclear surface towards the end of interphase stage (arrowheads, PCM, $\gamma$–tubulin; arrows, sperm axoneme). **B**: The mouse central anaphase mitotic spindle (green) is anastral with elongating, crisscrossing interzonal microtubules radiating toward the cortex as the chromosomes segregate to opposite poles (blue). **C**: As the chromosomes complete migration to opposite poles at telophase (blue), the spindle microtubules expand from the spindle apparatus (green). Cleavage furrow formation begins symmetrically in the opposing cortical regions and orthogonal to developing midbody (arrows). **D**: Enhanced myosin II (green) at telophase is observed at the leading edges of the

symmetrical invaginating cleavage furrow (arrows) as daughter cell nuclei reform (blue). A: triple-labeled for microtubules (green), acetylated $\alpha$-tubulin (red) and DNA; B and C: double-labeled for microtubules (green) and DNA (blue); D: double-labeled for myosin II (green) and DNA (blue). Bar=10$\mu$m.

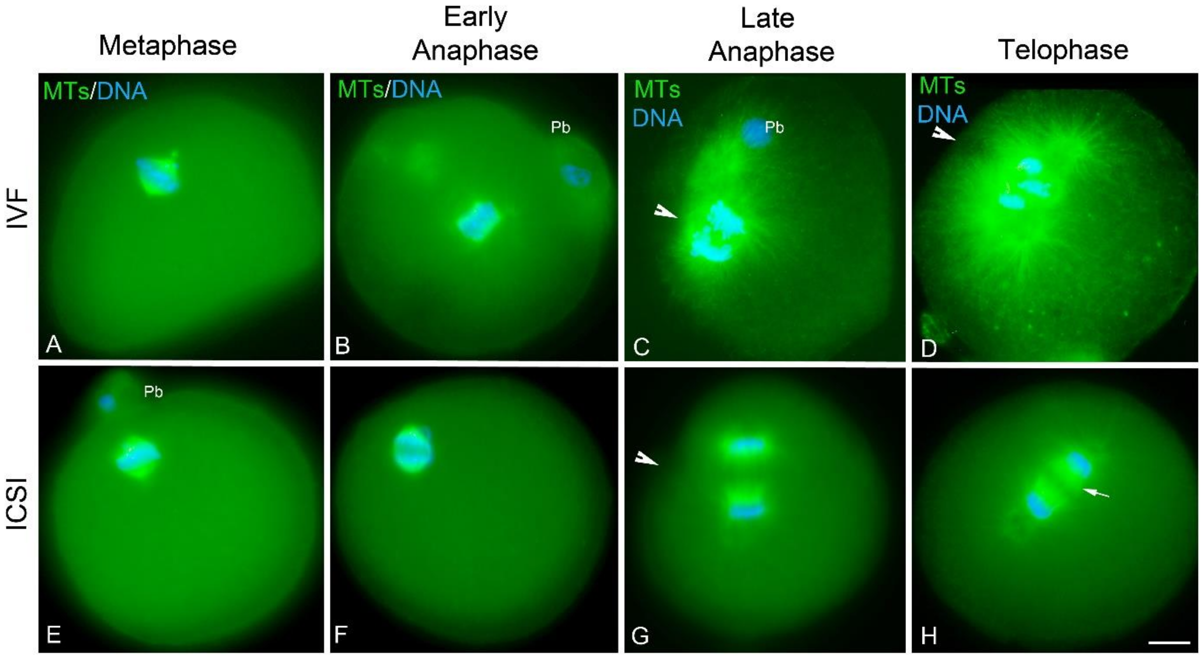


**Supplemental Figure S3. Random Orientation of the Interphase Pronuclei after ICSI Induces Off-Axis Mitotic Spindle Assembly and Unequal Cleavage Division Compared to IVF Zygotes.** (**A-D**): Representative images of mitotic IVF fertilized zygotes. At metaphase, an anastral bipolar spindle (A: green) forms para-tangential to the overlying cortex. Cleavage progression is orthogonal to the aligned equatorial chromosomes (A: blue), indicating equal daughter blastomere formation after cytokinesis. At early anaphase, as the sister chromatids separate (B: blue), the spindle poles broaden and start assembly of polar microtubules toward the cortex (B: green). Note orthogonal division plane through the separating sister chromatids would include passage through the 2$^{nd}$ polar body (B: Pb). By late anaphase, extensive spindle pole microtubules project toward the cortex (C: green) as the asymmetric cleavage furrow

begins to invaginate (C: arrowhead) orthogonal to the plane of chromosome separation (C: blue). D: a polyspermic IVF oocyte with 3 sets of mitotic chromosomes at early telophase (blue). Spindle pole microtubules (green) overlapping at the adjacent cortex signal the initiation of cleavage furrow assembly orthogonal to the plane of separating chromosomes (arrowhead). (**E-H**). Representative images of mitotic ICSI-fertilized zygotes. After ICSI fertilization, random apposed cortical pronuclear orientation positions centrosomes that assemble mitotic metaphase spindles with a radial cortical orientation (E: green; DNA, blue). At early anaphase, as sister chromatids separate (F: blue), no enhanced spindle pole microtubule assembly toward the cortical region is observed (F: green). The cleavage plane expected to pass orthogonal to the plane of separating chromosomes would produce unequally sized daughter blastomeres post-cytokinesis. With advancing anaphase (G: blue, DNA), sparse spindle pole microtubules radiate cortically (G: green) as the asymmetric cleavage furrow begins to progress orthogonal to the separating chromosomes. Unequal daughter cell formation is expected as the furrow plane advances. H: an ICSI zygote with a central cytoplasmic telophase spindle having well-separated chromosomes (blue), elaborated spindle pole microtubules (green), and interzonal microtubules forming the midbody (arrow). Note the late onset of cleavage furrow initiation at this advanced mitotic stage. All images are double-labeled for microtubules (green) and DNA (blue). Bar= 20µm.

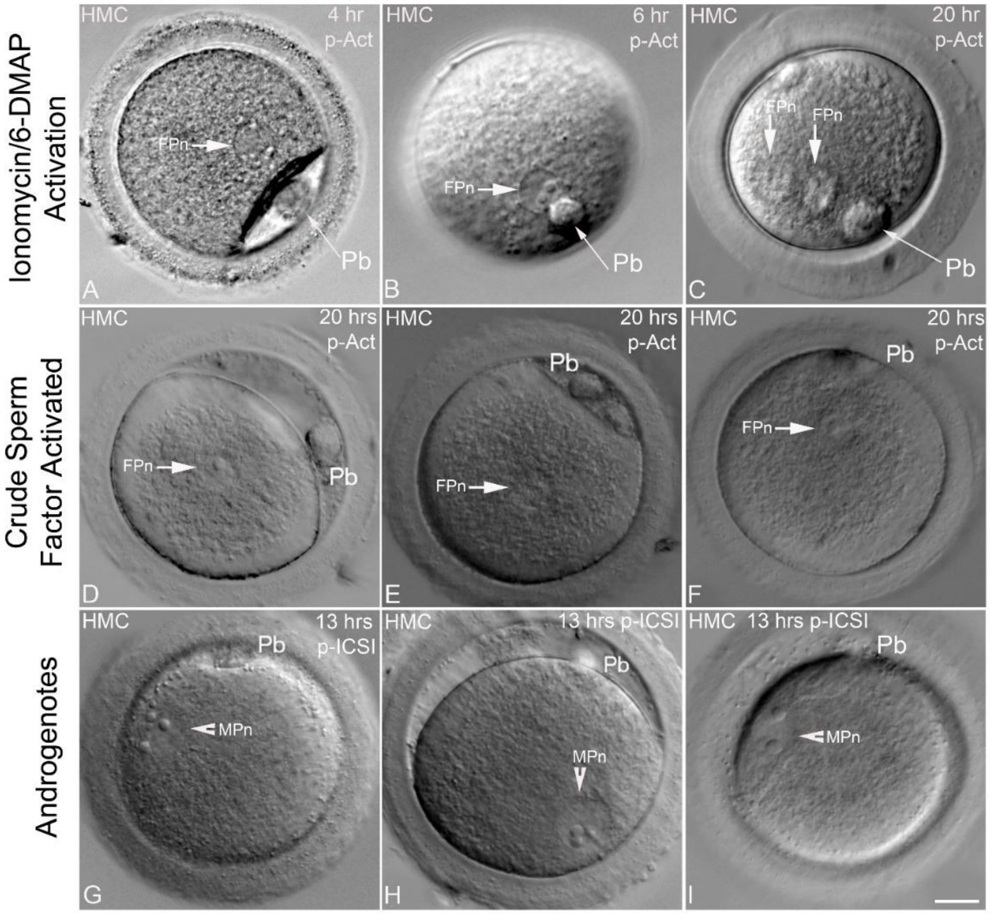


**Supplemental Figure S4. Eccentric Pronuclear Orientation in NHP Zygotes is Unique to the Male Pronucleus.** (**A-C)**: Three examples of pronuclear-stage parthenogenotes (no male contribution) produced by artificial activation in 5µM ionomycin for 5 min, followed by 4hr in 1.9mM 6-DMAP exposure. At the end of 6-DMAP treatment, often a single diploid female pronucleus (FPn) forms in the cytoplasm, initially at the site of the second meiotic spindle (A-B, short arrows). Rarely, two haloid FPn from the product of meiosis-II completion, without the elicitation of the second polar body, forms. The FPn formed in parthenogenotes often drift toward the central cytoplasm after activation and before the onset of first mitosis (C: arrows). The polar bodies (Pb) in A-C (long arrows) represents the products from completion of first meiosis. (**D-F**): Rhesus parthenogenotes derived by injection of a crude sperm factor into mature oocytes. Unlike ionomycin/6-DMAP activation, injection of a crude sperm extract

triggers the completion of second meiosis with the elicitation of the second polar body (Pb). By 20 hrs post-activation, the single female pronucleus formed in each zygote (arrows) drifts away from the cortical region toward the central cytoplasm, where spindle assembly occurs following nuclear envelop breakdown. (**G-I**): Three examples of pronuclear-stage androgenotes (no female nuclear contribution) formed by ICSI fertilization of enucleated oocytes produced when the female chromosomes and the second meiotic spindle apparatus were removed with a micropipette. The eccentric male pronuclei (arrows) tightly associate with the cortex by 13-hrs post-ICSI, unlike observations made in parthenogenetic zygotes. Hoffman Modulation Contrast (HMC) Imaging. Pb:1st or 2nd polar bodies. Bar=20μm.

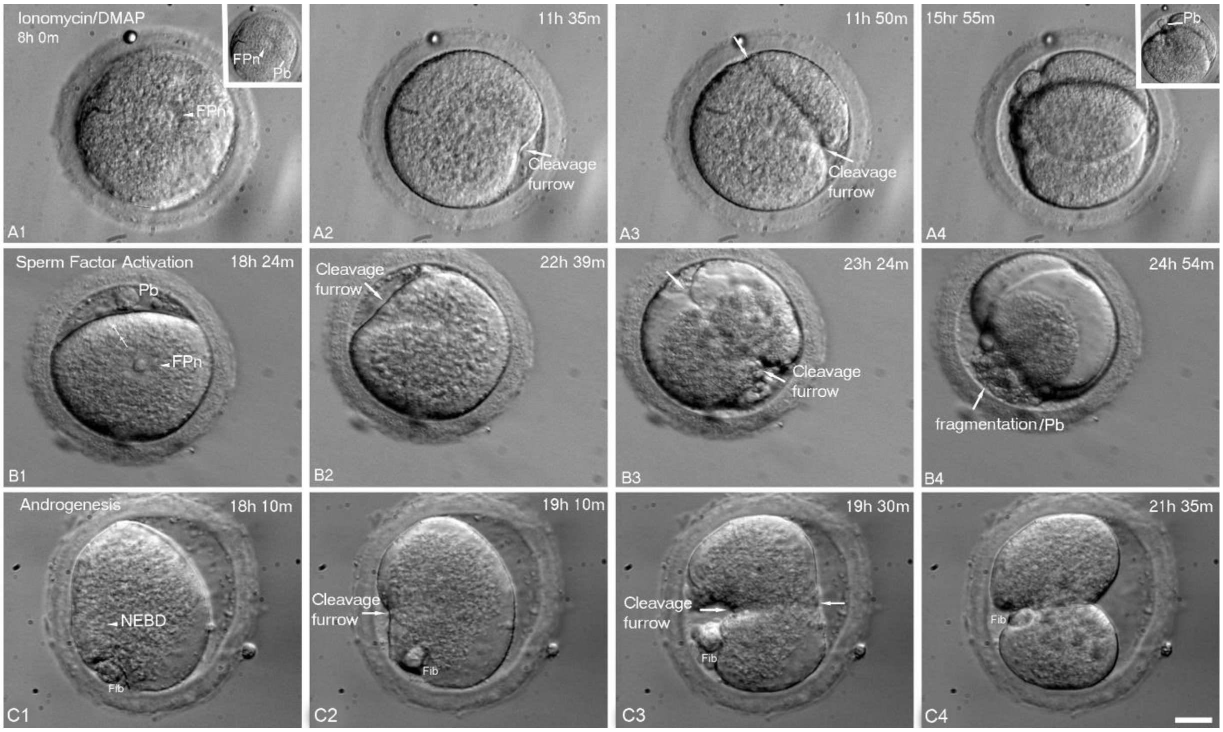


**Supplemental Figure S5. First Cleavage Patterns in Parthenogenetic and Androgenetic Zygotes.** (**A1-A4**): Diploid female parthenogenetic zygote (no 2nd polar body elicitation) created by chemical activation with ionomycin/6-DMAP. By 8hrs p-ICSI, the dual haploid female pronuclei have formed and migrated toward the central cytoplasm (A1; inset: 1st Pb). By 11.5 hrs p-ICSI, the central female pronuclei undergo nuclear envelope breakdown (NEBD) with the

formation of an asymmetric cleavage furrow at the distal cortex (A2: arrow). The cleavage furrow progresses to the opposite cortex (A3: arrows), forming roughly equal daughter blastomeres with slight fragmentation observed after cell division (A4; inset: 1st polar body at opposite cortex). (**B1-B4**): A haploid single pronuclear female parthenogenote (with 2nd polar body elicitation) after injection of NHP crude sperm factor. By 18.5 hrs p-ICSI, the central cytoplasmic female pronucleus quickly migrates toward the cortex, where the 2nd polar body (B1: Pb) formed after oocyte activation (B1: arrowhead, FPn; small arrows: direction of pronuclear movement). By ~22.5 hrs p-ICSI, the female pronucleus undergoes NEBD as the asymmetric cleavage furrow begins at the adjacent cortex (B2: arrow). As cleavage progresses (B3: arrows), cytoplasmic “clearing,” fragmentation, and a massive cortical rotation ensue, with the Pb and cytoplasmic fragments located between equal-sized daughter blastomeres (B4, arrow). (**C1-C4**): Haploid androgenote produced by ICSI after “enucleation” of the second meiotic spindle and 1st polar body. A fibroblast cell (Fib) inserted under the zona pellucida failed to fuse in this somatic cell nuclear transfer attempt. By 18 hrs, the eccentric cortical male pronucleus (MPn) undergoes NEBD cortically (C1: arrowhead). Cleavage furrow initiation begins at the oolemma overlying the former MPn site (C2: arrow) and progresses asymmetrically to the opposite cortex (C3: arrows). The non-fused fibroblast cell (C4: Fib) rotates into the cleavage furrow, residing between the daughter blastomeres after cytokinesis. All panels are Hoffman Modulation Contrast (HMC) images taken from TLVM movies of artificially activated parthenogenetic oocytes (A and B) or androgenotes derived by ICSI of oocytes devoid of maternal chromatin. Time in hrs:min (h:m) post-ICSI (p-ICSI). Bar=20μm.

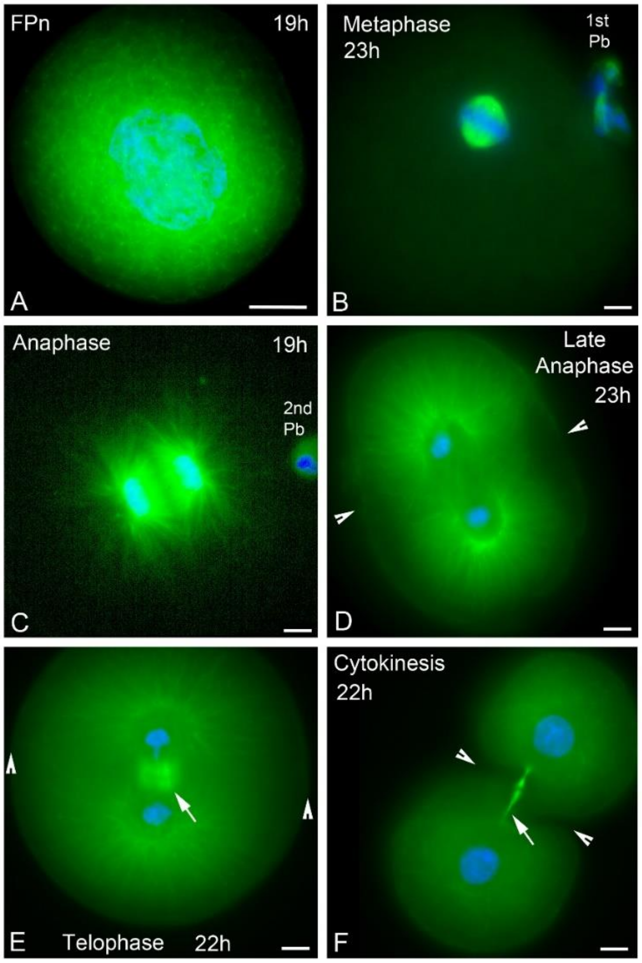


**Supplemental Figure S6. Microtubule and DNA Patterns in Parthenogenetic Zygotes.** (**A**): Interphase parthenogenotes generated using cytoplasmic injection of a crude sperm factor into a metaphase II-arrested rhesus oocyte. By 19hrs post-sperm factor activation, random cytoplasmic microtubule patterns assemble in the cytoplasm around the centralized female pronucleus without any paternal centriole contribution (A: green). (**B**): A parthenogenetic zygote produced by chemical activation with ionomycin/6-DMAP assembles short, anastral bipolar spindles (green, microtubules) with aligned chromosomes (blue, DNA) in the central cytoplasm by 23hrs post activation. Pb: the first polar body undergoing division. (**C**): A parthenogenetic zygote at mitotic anaphase produced 19hrs post-activation after microinjection of crude sperm factor. As the duplicated chromatids segregate toward their respective spindle poles (blue), short microtubules extend from the anastral bipolar spindle, crisscrossing at the spindle equator

but not extending to the distal cortex (green, microtubules), to signal asymmetric cleavage furrow initiation. Pb: 2nd polar body. (**D**): A 22hrs-post-activation late anaphase parthenogenetic zygote produced after crude sperm factor microinjection. As the separated female chromatids reach their respective spindle poles (blue), microtubule assembly increases dramatically from both spindle poles (green), with some radiating toward distal cortical regions. Note overlapping microtubules in the plane directly orthogonal to the spindle equatorial region but the paucity of microtubules reaching the cortical region, upon which the cleavage furrow is expected to assemble (arrowheads). (**E**): A 23hrs-post-activation parthenogenetic telophase zygote produced by crude sperm factor microinjection. As the separated female chromosomes begin to decondense into daughter nuclei (blue), microtubules radiate from the spindle poles (green) but do not contact the cortex, where cleavage furrow initiation (arrowheads) is predicted to form orthogonal to the assembling midbody (arrow). (**F**): By 22 hrs post-activation, a crude sperm factor-activated zygote completes cytokinesis, showing daughter cell nuclei reconstitution (blue) and greatly reduced spindle pole microtubules (green) as the equal-cleavage furrow invaginates orthogonal to the well-formed midbody (arrow). Pb: 1st or 2nd polar bodies. Time post-activation in hrs:min (h:m). All images double-labeled for microtubules (green) and DNA (blue). Bars=20μm.

**Supplemental Table 1. Comparison of Fertilization and Cytokinesis Events in Mice, Nonhuman Primates (NHP) and Human Zygotes.**

| Event | Mice | NHP (macaques, baboon) | Human | Selected References |
|---|---|---|---|---|
| **Metaphase-II Oocyte polarity** | Yes | unknown | Yes | *Mice*: rev Ajduk et Zernicka-Goetz, 2016; Chaigne et al, 2017<br>*Human*: Antczak and Van Blerkom, 1997; Van Blerkom et al, 2008; Garello et al, 1999; Coticchio et al, 2014<br>*alternative view: Motosugi et al, 2006 |
| **Sperm Incorporation Cone** | Yes | No | Yes | *Mice*: Longo, 1988; Simerly et al, 1998; Terada et al, 2000; *NHP*: Piotrowska-Nitsche et al, 2009; this report<br>*Human:* Iwata et Mio, 2016; Coticchio et al, 2018 |
| **Inherited sperm centrioles** | No (maternal acentriolar MTOCs) | Yes | Yes | *Mice*: Maro et al, 1985; Schatten et al, 1986; rev, Schatten, 1994; *NHP*: Wu et al, 1996; Hewitson et al, 1997<br>*Human*: Sathananthan et al,1991; Simerly et al, 1995; Van Blerkom et al, 1995 |
| **Sperm aster assembly for directed pronuclear migration** | No | Yes | Yes | *Mice*: Schatten et al, 1985; Schatten et al, 1986; Maro et al, 1986; *NHP*: Wu et al, 1996; Hewitson et al, 1997; *Human:* Simerly et al, 1995; Van Blerkom et al, 1995; Van Blerkom, 1996; Coticchio et al, 2018 |
| **Zygote shape change** | Yes | Yes | Yes | *Mice:* Grey et al, 2004; *NHP:* this study<br>*Human:* Payne et al, 1997; Coticchio et al, 2018 |
| **Cytoplasmic streaming/wave** | Yes (streaming) | Yes (streaming/wave) | Yes (wave) | *Mice:* Deguchi et al, 2000; Ajduk et al, 2011; Lee et al, 2013; Almonacid et al, 2015; Niwayama et al, 2016; *NHP:* this study; *Human:* Payne et al, 1997; rev, Scott, 2000; Swann et al, 1997; Coticchio et al, 2018. |
| **Pronuclear position at end of interphase** | central | eccentric | central | *Mice:* Schatten et al, 1986; Maro et al, 1985; *NHP*: Wu et al, 1996; *Human:* Simerly et al, 1995; Van Blerkom et al, 1995; Payne et al, 1997; Scott, 2000; Coticchio et al, 2018 |
| **Mitotic spindle assembly** | Maternal acentriolar MTOCs | Duplicated sperm centrioles | Duplicated sperm centrioles | *Mice:* Schatten et al, 1986; Maro et al, 1986; *NHP:* Wu et al, 1996; *Human:* Santhanthan et al, 1991; Simerly et al, 1995; Van Blerkom et al,1996. |
| **Cleavage furrow initiation** | symmetric | asymmetric | symmetric | *Mice*: Schatten et al, 1986; Simerly et al, 1998; Maro et al, 1985; *NHP*: this study<br>*Human:* Simerly et al, 1995; Coticchio et al, 2018. |
| **Cleavage furrow perpendicular to aligned parental genomes** | Yes* | Yes | unknown | *Mice:* Hiiragi et Solter, 2004; *NHP:* this study; *Human:* not reported<br>*alternative view: Plusa et al, 2005 |
| **Cleavage furrow through animal axis (within 30° of animal axis [polar body])** | IVF: Yes*<br>ICSI: unk | IVF: unk<br>ICSI: Yes | IVF: unk<br>ICSI: Yes | *Mice*: Gray et al, 2004; Plusa et al, 2002;2005; *NHP*: this study; *Human:* Desai et al, 2019.<br>*alternative views: Hiiragi et Solter, 2004 |
| **Cleavage furrow through sperm entrance point** | IVF: Yes*<br>ICSI: unk | IVF: unk<br>ICSI: No | IVF: unk<br>ICSI: unk | *Mice*: Piotrowska et Zernicka-Goetz, 2001; Plusa et al, 2002; Gray et al, 2004; Plusa et al, 2005; *NHP*: Piotrowska-Nitsch et al, 2008; *Human:* Mio and Maeda, 2008<br>*alternative views: Davies et Gardner, 2002; Hiiragi et Solter, 2004. |

**SUPPLEMENTAL VIDEOS.**

**Supplemental Video 1.** Selected time-lapse video frames from a recording of Rhesus sperm incorporation during *in vitro* fertilization. The sperm head is bound to the oolemma by 3hrs post-insemination (blue arrow). The oocyte's cortex initially rotates toward the middle oocyte area, the site of eventual 2$^{nd}$ polar body elicitation. Note the cytoplasm "expands" outward as oocyte activation ensues, carrying the bound sperm head toward the far upper left cortical region. This is an example of the observed oocyte shape changes during fertilization. The female pronucleus (out of the plane of focus) forms after 2$^{nd}$ polar body extrusion and migrates to the cortical decondensing male sperm head (pink arrow, FPn). Video is 9 frames taken from an 8.5-hr multidimensional TLVM recording at 5 min per frame.

**Supplemental Video 2.** Split-screen video of Rhesus *in vitro* fertilization, with female pronuclear events observed on the left beginning after 2$^{nd}$ polar body elicitation (pink arrow, FPn), and male pronuclear events beginning with the attached incorporated sperm head seen on the right (blue arrow, MPn). The female pronucleus migrates to the male pronucleus on the assembled sperm astral microtubules while the male sperm head undergoes decondensation and pronuclear formation near the cortex. After female pronuclear migration, the apposed pronuclei orient radially to the overlying cortex, with the female pronucleus distal to the cortical-residing male pronucleus. Each video is 11 frames taken from a 16-hr multidimensional TLVM recording at 15 min per frame.

**Supplemental Video 3.** Split-screen video of Rhesus ICSI fertilization beginning 3hrs post-sperm microinjection, with female pronuclear events observed on the left and male pronuclear events on the right. Polar body elicitation occurs by 5hrs post-ICSI, with female pronuclear assembly (left: pink arrow, FPn) followed by migration to the cortical decondensing male pronucleus (right: blue arrow, MPn). The microinjected sperm head glides extensively along the cortex prior to male pronuclear decondensation (right: blue arrow, sperm head). After

pronuclear apposition, the pronuclei align para-tangentially to the overlying cortex as opposed to radial orientation observed after IVF (compare to Supplemental Video 2). Each video is 17 frames taken from a 15-hr multidimensional TLVM recording at 15 min per frame.

**Supplemental Video 4.** Rhesus ICSI multidimensional TLVM beginning 6.5hrs post-sperm injection. The video shows female pronuclear (pink arrow, FPn) migration to the cortical male pronucleus (blue arrow, MPn), radial pronuclear orientation and meridional cleavage, which begins near the formerly intact male pronucleus that proceeds through the 2$^{nd}$ polar body region (Pb). Video is 14 selected frames from a 24-hr TLVM recording with images taken every 10 min.

**Supplemental Video 5.** Cynomolgus ICSI multidimensional TLVM recording beginning 3.5hrs post-sperm injection. The video shows 2$^{nd}$ polar body elicitation, female pronuclear formation (pink arrow, FPn), and migration to the cortical-residing male pronucleus (blue arrow, MPn). The apposed pronuclei are para-tangentially arranged to the cortex, with cleavage furrow initiation beginning near the formerly intact male pronucleus (arrow) and progressing equatorially to the opposite cortex as the polar bodies rotate into the cleavage furrow (1$^{st}$ polar body; 2$^{nd}$ polar body). Video is 14 frames selected from a 24-hr recording with image capture every 10 min until mitosis, when recordings are switched to 2 min per image capture.

**Supplemental Video 6.** Baboon ICSI multidimensional TLVM recording beginning 11.5hrs post-sperm injection. The apposed male (blue arrow, MPn) and female (pink arrow, FPn) pronuclei are oriented radially to the overlying cortex. Two PHA-coated fluorescent beads are attached to the outside oolemma, a large 5-µm bead marking the site of ICSI (white arrow, MIP) and a smaller 3-µm bead labeling an off-target cortical site (green arrow, cortical fluorescent bead). Female pronuclear breakdown occurs before male pronuclear breakdown, and the cleavage furrow initiates near the site of the formerly intact male pronucleus (white arrow, cleavage initiation), progressing meridionally to the opposing cortex, where the polar bodies

(Pbs) reside, but not through either cortical site marked by the attached fluorescent beads. Cytokinesis forms unequal-sized daughter blastomeres, with the polar bodies between the daughter cells and both fluorescent beads rotating toward the furrow region.  Video is 14 frames taken from a 20-hr recording with frames taken every 10 minutes.

**Supplemental Video 7.** Baboon ICSI multidimensional TLVM recording beginning 10 min post-ICSI.  The video shows 2$^{nd}$ polar body elicitation, female pronuclear formation (pink arrow, FPn), and migration to the cortical male pronucleus (blue arrow, MPn).  The apposed pronuclei briefly separate and then re-establish contact as they tumble along the cortical region prior to pronuclear envelope breakdown, with the female pronucleus nearest the overlying cortex at mitosis onset.  The cleavage furrow begins near the site of the formerly intact female pronucleus and proceeds meridionally to the opposing cortex through the 2$^{nd}$ polar body region in an unequal cleavage pattern.  A 3-μm fluorescent bead marks the site of ICSI at the microinjection point (MIP) but detaches as the pronuclear migration occurs and the zygote cytoplasm contracts.  As mitosis begins, the cytoplasm expands and re-engages the fluorescent bead, which rotates toward the cleavage furrow.  Note, neither cleavage initiation nor progression includes the cortical site marked with a fluorescent bead.  Both blastomeres undergo equatorial cleavages at second division.  Video is 14 frames taken from a 40.5-hr recording with frames taken every 10 min.

**Supplemental Video 8**.  Split-screen videos of Rhesus oocyte ionomycin/6-DMAP parthenogenetic activation (left) and androgenesis (right) from an “enucleated” oocyte that failed to fuse with a somatic cell (somatic cell nuclear transfer failure).  Parthenogenetic activation begins in late interphase with two haploid female pronuclei (pink arrows, FPN) visible in the cytoplasm distal to the 1$^{st}$ polar body.  Following simultaneous female pronuclear breakdown, the cleavage furrow (arrow) begins near the site of the 1$^{st}$ polar body (not visible in focal plane) and moves to the opposing cortical site.  The first polar body resides at the opposite cortex

following migration along the cleavage furrow during cytokinesis. In the androgenote (right video), the oocyte was first enucleated to remove the second meiotic spindle apparatus and the first polar body; this was followed by ICSI. A somatic cell was inserted under the zona pellucida after ICSI and an electric current applied to fuse with the enucleated oocyte, but this failed. The ICSI oocyte formed a single haploid male pronucleus near the cortex (blue arrow, MPn) by 18.5hrs post-sperm injection, with cleavage furrow formation near the formerly intact male pronucleus and progressing to the opposing cortex. The bound somatic cell rotates in to the cleavage furrow region during cytokinesis. The parthenogenote video is 8 frames selected from a 12-hr TLVM recording with image capture every 5 min. The androgenote video is 8 frames selected from a 4-hr recording with image capture every 5 min.

**REFERENCES.**


1. Ajduk, A. & Zernicka-Goetz, M. Polarity and cell division orientation in the cleavage embryo: from worm to human. *Mol. Hum. Reprod.* **22**(10):691-703 (2016).

2. Antczak, M. & Van Blerkom, J. Oocyte influences on early development: the regulatory proteins leptin and STAT3 are polarized in mouse and human oocytes and differentially distributed within the cells of the preimplantation stage embryo. *Mol. Hum. Reprod.* **3**(12):1067-86 (1997).

3. Van Blerkom, J., Davis, P. & Thalhammer, V. Regulation of mitochondrial polarity in mouse and human oocytes: the influence of cumulus derived nitric oxide. *Mol. Hum. Reprod*.**14**, 431-44 (2008).

4. Garello, C., Baker, H., Rai, J., Montgomery, S., Wilson, P., Kennedy, C.R. & Hartshorne, G.M. Pronuclear orientation, polar body placement, and embryo quality after intracytoplasmic sperm injection and in-vitro fertilization: further evidence for polarity in human oocytes? *Hum. Reprod.* **14**(10):2588-95 (1999).

5. Coticchio, G., Guglielmo, M.C., Albertini, D.F., Dal Canto, M., Mignini Renzini, M., De Ponti, E. & Fadini, R. Contributions of the actin cytoskeleton to the emergence of polarity during maturation in human oocytes. *Mol. Hum. Reprod.* **20**(3):200-7. doi: 10.1093/molehr/gat085. Epub 2013 Nov 20. (2014).

6. Longo, F.J. Reorganization of the egg surface at fertilization. *Int. Rev. Cytol.* **113**:233-69 (1988).

7. Simerly, C., Nowak, G., de Lanerolle, P. & Schatten, G. Differential expression and functions of cortical myosin IIA and IIB isotypes during meiotic maturation, fertilization, and mitosis in mouse oocytes and embryos. *Mol. Biol. Cell* Sep;**9**(9):2509-25 (1998).

8. Terada, Y., Simerly, C. & Schatten, G. Microfilament stabilization by jasplakinolide arrests oocyte maturation, cortical granule exocytosis, sperm incorporation cone resorption, and cell-cycle progression, but not DNA replication, during fertilization in mice. *Mol. Reprod. Dev.* **56**(1):89-98 (2000).

9. Piotrowska-Nitsche, K., Yang, S.H., Banta, H. & Chan, A.W. Assisted fertilization and embryonic axis formation in higher primates. *Reprod. Biomed. Online* **18,** 382-390 (2009).

10. Iwata, K. & Mio, Y. Observation of human embryonic behavior in vitro by high-resolution time-lapse cinematography. *Reprod. Med. Biol*. **15,** 145-154 (2016).

11. Coticchio, G., Mignini, R., Novara, P.V., Lain, M., De Ponti, E., Turchi, D., Fadini, R. & Dal Canto, M. Focused time-lapse analysis reveals novel aspects of human fertilization and suggests new parameters of embryo viability. *Hu. Reprod.* **33**(1):23-31 (2018).

12. Maro, B. Fertilization and the cytoskeleton in the mouse. *Bioessays* **3**(1):18-21 (1985).

13. Schatten, H., Schatten, G., Mazia, D., Balczon, R. & Simerly, C. Behavior of centrosomes during fertilization and cell division in mouse oocytes and in sea urchin eggs. *Proc. Natl. Acad. Sci. USA.* **83**(1):105-9 (1986).

14. Schatten, G. The centrosome and its mode of inheritance: the reduction of the centrosome during gametogenesis and its restoration during fertilization. *Dev. Biol.* **165,** 299-335 (1994).

15. Wu, G-J., Simerly, C., Zoran, SS., Funte, L.R. & Schatten, G. Microtubule and chromatin dynamics during fertilization and early development in rhesus monkeys and regulation by intracellular calcium ions. *Biol. Reprod.* **55,** 260-270 (1996).

16. Hewitson, L., Simerly, C. & Schatten, G. Inheritance defects of the sperm centrosome in humans and its possible role in male infertility. *Int. J. Androl.* **20,** Suppl 3:35-43 (1997).

17. Sathananthan, A.H., Kola, I, Osborne, J, Trounson, A, Nga, SC, Bongso, A & Ratnam, SS. Centrioles in the beginning of human development. *Pro. Natl. Acad. Sci. USA* **88,** 4806-4810 (1991).

18. Simerly, C., Wu, G.J., Zoran, S., Ord, T., Rawlins, R., Jones, J., Navara, C., Gerrity, M., Rinehart, J., Binor, Z., Asch, R. & Schatten, G. The paternal inheritance of the centrosome, the cell's microtubule-organizing center, in humans, and the implications for infertility. *Nat. Med*. Jan;1(1):47-52. Erratum in: *Nat. Med*. Jun;**1**(6):599 (1995).

19. Van Blerkom, J., Davis, P., Merriam, J. & Sinclair, J. Nuclear and cytoplasmic dynamics of sperm penetration, pronuclear formation and microtubule organization during fertilization and early preimplantation development in the human. *Hum. Reprod*. **1**(5):429-61 (1995).

20. Schatten, G., Simerly, C. & Schatten, H. Microtubule configurations during fertilization, mitosis, and early development in the mouse and the requirement for egg microtubule-mediated motility during mammalian fertilization. *Proc. Natl. Acad. Sci. USA.* **82**(12):4152-6 (1985).

21. Schatten, G., Simerly, C. & Schatten, H. Microtubules in mouse oocytes, zygotes, and embryos during fertilization and early development: unusual configurations and arrest of mammalian fertilization with microtubule inhibitors. *Ann. N. Y. Acad. Sci*. **466,** 945-8 (1986).

22. Maro, B., Howlett, S.K. & Houliston, E. Cytoskeletal dynamics in the mouse egg. *J. Cell Sci. Suppl.* **5,** 343-59 (1986).

23. Van Blerkom, J. Sperm centrosome dysfunction: a possible new class of male factor infertility in the human. *Mol. Hum. Reprod.* **2**(5):349-54 (1996).

24. Gray, D., Plusa, B., Piotrowska, K., Na, J., Tom, B., Glover, D.M. & Zernicka-Goetz, M. First cleavage of the mouse embryos responds to change in egg shape at fertilization. *Curr. Biol.* **14,** 397-405 (2004).

25. Payne, D., Flaherty, S.P., Barry, M.F. & Mathews, C.D. Preliminary observations on polar body extrusion and pronuclear formation in human oocytes using time-lapse video cinematography. *Hum. Reprod.* **12,** 532-541 (1997).

26. Deguchi, R., Shirakawa, H., Oda, S., Mohri, T. & Miyazaki, S. Spatiotemporal analysis of Ca (2+) waves in relation to the sperm entry site and animal-vegetal axis during Ca (2+) oscillations in fertilized mouse eggs. *Dev. Biol.* **218**, 299-313 (2000).

27. Ajduk, A., Ilozue, T., Windsor, S., Yu, Y., Seres, K.B., Bomphrey, R.J., Tom, B.D., Swann K, Thomas A, Graham C & Zernicka-Goetz M. Rhythmic actomyosin-driven contractions induced by sperm entry predict mammalian embryo viability. *Nat Commun*. **2,**417, doi: 10.1038/ncomms1424 (2011).

28. Lee, S.Y., Lee, H.S., Kim, E.Y., Ko, J.J., Yoon, T.K., Lee, W.S. & Lee, K.A. Thioredoxin-interacting protein regulates glucose metabolism and affects cytoplasmic streaming in mouse oocytes. *PLoS One.* **8**(8):e70708. doi: 10.1371/journal.pone.0070708. eCollection (2013).

29. Almonacid, M., Ahmed, W.W., Bussonnier, M., Mailly, P., Betz, T., Voituriez, R., Gov, N.S. & Verlhac, M.H. Active diffusion positions the nucleus in mouse oocytes. *Nat. Cell Biol.* **17**(4):470-9. doi: 10.1038/ncb3131. Epub 2015 Mar 16. (2015).

30. Niwayama, R., Nagao, H., Kitajima, T.S., Hufnagel, L., Shinohara, K., Higuchi, T., Ishikawa, T. & Kimura, A. Bayesian inference of forces causing cytoplasmic streaming in Caenorhabditis elegans embryos and mouse Oocytes. *PLoS ONE* **11**(7): e0159917. doi:10.1371/ journal.pone.0159917 (2016).

31. Scott, L. Pronuclear scoring as a predictor of embryo development. *Reprod. Biomed. Online* (2000).

32. Swann, K. & Lai, F.A. A novel signaling mechanism for generating $Ca^{2+}$ oscillations at fertilization in mammals. *B*ioessays **19**(5):371-378 (1997).

33. Maro, B., Howlett, S.K., & Webb, M. Non-spindle microtubule organizing centers in metaphase II-arrested mouse oocytes. *J. Cell Biol.* **101**(5 Pt 1):1665-72 (1985).

34. Hiiragi, T. & Solter, D. First cleavage plane of the mouse egg is not predetermined but defined by the topology of the two apposing pronuclei *Nature* **430,** 360−364 (2004).

35. Plusa, B., Hadjantonakis AK, Gray D, Piotrowska-Nitsche K, Jedrusik A, Papaioannou VE, Glover DM, & Zernicka-Goetz M. The first cleavage of the mouse zygote predicts the blastocyst axis. *Nature* **434,** 392-395 (2005).

36. Plusa, B., Grabarek, J.B., Piotrowska, K., Glover, D.M., & Zernicka-Goetz, M. Site of the previous meiotic division defines cleavage orientation in the mouse embryo. *Nature Cell Biol.* **4,** 811−815 (2002a).

37. Plusa, B., Piotrowska, K., & Zernicka-Goetz, M. Sperm entry position provides a surface marker for the first cleavage planes of the mouse zygote. *Genesis* **32,** 193-198 (2002b).

38. Desai, N. & Gill, P. Blastomere cleavage plane orientation and the tetrahedral formation are associated with increased probability of a good-quality blastocyst for cryopreservation or transfer: a time-lapse study. *Fert. Steril*. pii.S0015-0282(19)30130-X. doi: 10.1016/j.fertnstert.2019.12.019 (2019).

39. Piotrowska, K. & Zernicka-Goetz, M. Role for sperm in spatial patterning of the early mouse embryo. *Nature* **409,** 517−521 (2001).

40. Mio, Y. & Maeda, K. Time-lapse cinematography of dynamic changes occurring during in vitro development of human embryos. *Am. J. Obstet. Gynecol.* **199,** 660.e1-660.e5 (2008).

41. Davies, T.J. & Gardner, R.L. The plane of first cleavage is not related to the distribution of sperm components in the mouse. *Hum. Reprod.* **17,**2368-2379 (2002).
